# Albumin-Based Drug Delivery for Glioblastoma Treatment: Mechanistic Rationale, Preclinical Evidence, and Clinical Translation

**DOI:** 10.3390/cells15131180

**Published:** 2026-06-29

**Authors:** Myung Geun Song, Keon Wook Kang

**Affiliations:** 1 Department of Nuclear Medicine, Seoul National University College of Medicine, Seoul 03080, Republic of Korea; mgsong0310@snu.ac.kr; 2 Biomedical Research Institute, Seoul National University Hospital, Seoul 03080, Republic of Korea; 3 Department of Biomedical Sciences, Seoul National University Graduate School, Seoul 03080, Republic of Korea; 4 Cancer Research Institute, Seoul National University College of Medicine, Seoul 03080, Republic of Korea

**Keywords:** glioblastoma, albumin nanoparticles, nab-paclitaxel, blood–brain barrier, blood–tumor barrier, focused ultrasound, translational nanomedicine

## Abstract

Glioblastoma remains the most aggressive primary brain malignancy, with poor survival despite maximal safe resection, radiotherapy, and temozolomide-based chemotherapy. A major obstacle to effective treatment is the spatially heterogeneous blood–brain barrier/blood–tumor barrier, which restricts drug penetration into infiltrative tumor regions and limits uniform intratumoral exposure. Albumin-based delivery is attractive in glioblastoma because it addresses several formulation-level barriers at once: poor aqueous solubility of hydrophobic payloads, short systemic exposure, and the need for a biocompatible carrier that can interact with albumin-handling pathways such as gp60/albondin, SPARC, FcRn, and caveolin-associated transport. This review examines albumin-based strategies explored for glioblastoma, with an emphasis on albumin-bound paclitaxel nanoparticles, engineered albumin nanoparticles, dual-payload systems, albumin-binding photosensitizers, macrophage-assisted delivery, and albumin-bound pathway-directed agents. Preclinical evidence suggests that these platforms can improve brain-tumor drug exposure, support rational combinations, and synergize with BBB/BTB-opening technologies. Early clinical studies combining low-intensity pulsed ultrasound with microbubbles and albumin-bound paclitaxel provide human proof of concept for regional pharmacokinetic enhancement in recurrent glioblastoma, although survival benefit remains unproven. The available evidence supports albumin-based delivery as a rational formulation strategy. Its clinical value in GBM will depend on three testable requirements: spatial pharmacokinetic confirmation, biomarker-guided patient selection, and reproducible BBB/BTB modulation.

## 1. Introduction

Glioblastoma (GBM) is classified in the 2021 World Health Organization (WHO) classification as glioblastoma, Isocitrate dehydrogenase (IDH)-wildtype, Central nervous system (CNS) WHO grade 4, and represents the most aggressive adult-type diffuse glioma of the central nervous system [1]. The current standard treatment backbone consists of maximal safe surgical resection followed by radiotherapy with concomitant and adjuvant temozolomide, a regimen that established a median overall survival of 14.6 months in the pivotal European Organisation for Research and Treatment of Cancer–National Cancer Institute of Canada (EORTC–NCIC) trial [2]. Despite this benchmark, durable disease control remains uncommon because glioblastoma is characterized by diffuse invasion, molecular heterogeneity, adaptive treatment resistance, and a profoundly immunosuppressive tumor microenvironment [3]. Population-based registry data from Central Brain Tumor Registry of the United States (CBTRUS) continue to show that glioblastoma imposes a major clinical burden across real-world patient cohorts, underscoring the need for therapeutic strategies that improve drug delivery without exacerbating neurologic toxicity [4].

The blood–brain barrier (BBB) and blood–tumor barrier (BTB) impose related but biologically distinct constraints on glioblastoma pharmacotherapy. In this review, the BBB refers to the relatively intact neurovascular barrier of normal brain parenchyma and infiltrative tumor margins, characterized by tight junctions, low paracellular permeability, efflux-transporter activity, pericyte support, and astrocytic regulation [5]. The BTB refers to the tumor-associated vascular interface within glioblastoma, where barrier integrity is variably disrupted by angiogenesis, tumor–vascular remodeling, and inflammatory signaling; this disruption is spatially heterogeneous and is usually more pronounced in contrast-enhancing tumor regions than in diffusely infiltrative margins [6,7]. This distinction is clinically important because increased permeability in the enhancing tumor core does not guarantee therapeutically meaningful exposure in peritumoral brain or invasive margins, where residual tumor cells frequently remain protected by relatively preserved BBB function [7].

For this review, “albumin-based drug delivery” refers to a broad class of therapeutic strategies in which albumin is used as a drug carrier, binding scaffold, formulation matrix, or biologically interactive transport component. This category includes clinically established albumin-bound drugs such as nanoparticle albumin-bound paclitaxel (nab-paclitaxel), engineered albumin nanoparticles, albumin-binding prodrugs or photosensitizers, albumin-functionalized or ligand-modified nanocarriers, albumin-bound pathway-directed agents, and cell-mediated strategies in which albumin-drug formulations are loaded into migratory immune cells [8,9,10]. These platforms are relevant to GBM because they can address formulation-level barriers, including poor aqueous solubility of hydrophobic payloads, short systemic exposure, and the need to coordinate systemic pharmacokinetics with controlled BBB/BTB access [9,10].

Albumin is biologically attractive as a carrier because it combines high plasma abundance, neonatal Fc receptor (FcRn)-mediated systemic persistence, broad hydrophobic ligand-binding capacity, biodegradability, and compatibility with nanoparticle assembly or chemical functionalization [8,9,11]. In addition, albumin can interact with glycoprotein 60 (gp60)/albondin, secreted protein acidic and rich in cysteine (SPARC), FcRn, and caveolin-associated pathways, thereby allowing carrier behavior to be influenced by endothelial transport, recycling, and tumor retention mechanisms under specific biological conditions [12]. These properties should not be interpreted as inherent proof of safety or tumor selectivity. Aggregation, albumin modification, residual impurities, immunogenicity, altered biodistribution, payload toxicity, and product-specific chemistry, manufacturing, and controls attributes remain central determinants of translational feasibility [8,10,13].

This review differs from previous reviews on GBM nanomedicine, BBB modulation, albumin nanoparticles, and nab-paclitaxel by focusing on whether albumin-associated carrier biology can be converted into clinically meaningful spatial drug exposure in human glioblastoma. Specifically, we synthesize evidence across four linked dimensions: albumin-specific transport and retention biology; spatial pharmacokinetic barriers created by BBB-preserved invasive margins and heterogeneous BTB permeability; integration of albumin-compatible therapeutics with BBB/BTB-opening technologies; and translational go/no-go criteria for clinical development, including tumor-to-normal brain exposure, invasive-margin penetration, receptor-pathway validation in human tissue, neurotoxicity, neurocognitive safety, and evidence that increased exposure translates into radiographic response or survival benefit [7,10,14].

### Search Strategy and Selection Criteria

This narrative review was prepared through a structured literature search of PubMed, Web of Science, ClinicalTrials.gov, and relevant reference lists. The search covered publications available up to 15 June 2026 and focused on studies related to albumin-based drug delivery, glioblastoma, high-grade glioma, blood–brain barrier (BBB), blood–tumor barrier (BTB), nab-paclitaxel, albumin nanoparticles, SPARC, gp60/albondin, FcRn, caveolin, focused ultrasound, microbubble-mediated BBB opening, convection-enhanced delivery, and translational nanomedicine. The following representative search terms and combinations were used: “glioblastoma” AND “albumin nanoparticle”; “glioblastoma” AND “nab-paclitaxel”; “albumin-bound paclitaxel” AND “blood–brain barrier”; “albumin” AND “SPARC” AND “glioma”; “gp60” OR “albondin” AND “albumin transcytosis”; “FcRn” AND “albumin drug delivery”; “focused ultrasound” AND “glioblastoma” AND “albumin-bound paclitaxel”; and “blood–tumor barrier” AND “nanomedicine”. Studies were included when they met at least one of the following criteria: direct evaluation of albumin-bound or albumin-interacting therapeutics in glioblastoma or high-grade glioma models; mechanistic assessment of albumin-associated transport, recycling, transcytosis, or tumor retention pathways relevant to CNS delivery; pharmacokinetic or biodistribution evidence involving brain, tumor, peritumoral tissue, or BBB/BTB modulation; clinical trial evidence involving albumin-bound therapeutics in brain tumors; or extracranial clinical evidence that provided formulation, dosing, safety, pharmacokinetic, manufacturing, or biomarker precedent relevant to GBM translation. Priority was given to peer-reviewed original studies, clinical trials, authoritative reviews, and clinical trial registry records when peer-reviewed efficacy data were not yet available.

Studies were excluded when they were unrelated to albumin-based delivery, lacked relevance to CNS or oncology drug delivery, focused only on non-albumin nanocarriers without a clear comparator or mechanistic link to albumin-based systems, or reported formulation concepts without sufficient characterization of payload loading, carrier design, biodistribution, biological mechanism, or therapeutic relevance. Preprints were not used as primary evidence. Non-peer-reviewed sources were considered only for clinical trial status, trial design, or regulatory context when no peer-reviewed publication was available. Because this article is a narrative critical review rather than a systematic review or meta-analysis, no formal risk-of-bias assessment, pooled quantitative synthesis, or Preferred Reporting Items for Systematic Reviews and Meta-Analyses (PRISMA)-based screening workflow was performed.

## 2. Properties of Albumin as a Drug Carrier

### 2.1. Structural and Biochemical Characteristics

Human serum albumin (HSA) is an approximately 66.5-kDa plasma protein with a flexible three-domain architecture that supports broad ligand binding, conformational adaptability, and interaction with endogenous and exogenous molecules [15]. Crystallographic studies indicate that HSA contains structurally adaptable drug-binding pockets capable of accommodating fatty acids, hormones, metabolites, and diverse small-molecule drugs [15]. This structural plasticity is directly relevant to drug delivery: hydrophobic domains can enhance the delivery of poorly water-soluble payloads, whereas surface-accessible amino, carboxyl, thiol, and lysine residues enable chemical conjugation, nanoparticle assembly, ligand functionalization, or albumin-binding prodrug design [8,9].

These biochemical features make albumin a versatile scaffold rather than a single delivery technology. Depending on formulation design, albumin can function as a solubilizing matrix, a covalent or noncovalent binding partner, a nanoparticle-forming protein, a depot for albumin-binding payloads, or a biologically interactive carrier capable of engaging albumin-handling pathways [9,10]. This versatility is particularly relevant to GBM because many therapeutically potent payloads are limited by poor solubility, short systemic exposure, or insufficient delivery to spatially protected tumor compartments [5].

### 2.2. Biocompatibility, Systemic Persistence, and Formulation-Dependent Safety

Albumin-based carriers are often described as biocompatible because albumin is a major plasma protein involved in the physiological transport of fatty acids, hormones, metabolites, and xenobiotics [9]. This biological familiarity supports its use as a drug-delivery scaffold, but it should not be interpreted as evidence that all albumin-based formulations are intrinsically safe. Biocompatibility depends on the specific product, including albumin source, degree of chemical modification, aggregation state, particle size distribution, residual solvent or impurity burden, payload toxicity, sterilization method, and chemistry, manufacturing, and controls (CMC) reproducibility [8,10,13].

A second major feature of albumin is its long systemic persistence. HSA has a plasma half-life of approximately 19 days, largely because neonatal Fc receptor (FcRn)-mediated recycling protects internalized albumin from lysosomal degradation [11,16,17]. After endocytic uptake, albumin binds FcRn in acidic endosomal compartments, avoids lysosomal degradation, and is recycled to the extracellular space at physiological pH [16,17]. For drug delivery, FcRn-mediated recycling may prolong systemic exposure and increase the probability that albumin-associated payloads encounter tumor vasculature; however, extended circulation alone does not ensure BBB/BTB crossing, invasive-margin penetration, intratumoral retention, or controlled payload release [10,11].

Albumin-based systems may also reduce reliance on some non-native scaffold components used in synthetic nanocarriers, such as polyethylene glycol (PEG) in PEGylated lipid or polymeric systems. Anti-PEG antibody responses and accelerated blood clearance have been reported for PEGylated liposomes, highlighting one reason why albumin-based constructs may be attractive for repeated dosing schedules [18]. This advantage should be framed cautiously. Albumin formulations can still trigger product-specific immunogenicity or toxicity when protein conformation is altered, particles aggregate, impurities remain, or the payload produces dose-limiting systemic or CNS toxicity [10,13]. In GBM, this distinction is especially important because BBB/BTB-opening strategies may increase CNS exposure while systemic toxicities of the payload remain relevant [14].

### 2.3. Albumin Receptor-Mediated Transport and Tumor Retention

The oncologic rationale for albumin-based delivery rests in part on albumin’s ability to engage endogenous handling pathways involved in endothelial transport, systemic recycling, and tumor-associated retention. In GBM, this rationale must be interpreted against the spatial heterogeneity of BBB and BTB permeability: contrast-enhancing tumor regions may show increased vascular permeability, whereas infiltrative margins and peritumoral brain often retain substantial BBB integrity [5,6,7]. Albumin-associated transport, therefore, offers a biologically plausible supplement to passive accumulation. However, its contribution is likely to depend on formulation design, vascular phenotype, albumin conformation, particle size, surface modification, receptor availability, and regional BBB/BTB status [10,12].

Glycoprotein 60, also known as gp60 or albondin, is an endothelial albumin-binding protein involved in caveola-associated albumin transcytosis [19]. Albumin–gp60 binding can induce receptor clustering, Src-family kinase activation, caveolin-1 phosphorylation, and caveolar vesicle formation, thereby supporting vesicular endothelial albumin transport in experimental endothelial systems [19,20]. In GBM-relevant albumin nanoparticle studies, gp60-associated transport has been used to explain BBB traversal and tumor vascular uptake. However, gp60 should be regarded as a vascular albumin-handling pathway rather than a GBM-selective targeting receptor [12,21].

Secreted protein acidic and rich in cysteine (SPARC) provides a complementary mechanism for tumor-associated albumin retention. SPARC is an albumin-binding matricellular glycoprotein that modulates cell–matrix interaction, collagen organization, stromal remodeling, growth-factor signaling, and vascular biology rather than functioning as a purely structural extracellular matrix protein [12,22]. Glioma-focused experimental systems have linked SPARC-associated interactions to albumin nanoparticle uptake and intratumoral retention; notably, SPARC knockout substantially reduced uptake of an albumin-binding photosensitizer in a glioma model [21,23]. These findings support SPARC as a mechanistically relevant albumin-retention axis in selected preclinical glioma contexts; at present, however, SPARC is better treated as a variable for delivery-mechanism studies than as a validated clinical predictor of response in GBM.

This distinction is important when albumin-based delivery is discussed as a targeting strategy. Gp60, SPARC, FcRn, and caveolin-associated pathways provide a useful biological framework for albumin-associated transport, systemic recycling, and retention. Still, clinical benefit from albumin-bound therapeutics in extracranial cancers has not been conclusively attributed to these pathways [12,22,24]. In an exploratory analysis of the phase III Metastatic Pancreatic Adenocarcinoma Clinical Trial (MPACT), SPARC expression did not predict the efficacy of nab-paclitaxel plus gemcitabine or gemcitabine alone in metastatic pancreatic cancer, reinforcing the need to treat SPARC as a context-dependent biological variable rather than a universal predictive biomarker [24]. Accordingly, gp60, SPARC, FcRn, and caveolin-associated pathways should be positioned as candidate mechanistic contributors and exploratory enrichment variables, not as established clinical predictors of response. In GBM, the translational value of these pathways should be tested prospectively through spatial pharmacokinetics, receptor-pathway profiling, patient-derived models, and tissue-level mapping of BBB/BTB heterogeneity [10,12]. Because these pathways overlap and vary by formulation, vascular context, and tumor region, Table 1 summarizes the principal mechanisms relevant to albumin-based GBM delivery and distinguishes albumin-associated biology from nonspecific BTB leakage or formulation-driven accumulation [7,10,12,25].

The mechanisms summarized in Table 1 are complementary and context-dependent, and several may operate simultaneously within the same formulation or tumor region [12,21]. This distinction is important because bulk tumor accumulation may reflect gp60/caveolin-associated endothelial trafficking, SPARC-associated retention, FcRn-mediated persistence, passive BTB leakage, or formulation-dependent release rather than a single albumin-specific transport mechanism [10,24]. Therefore, mechanistic attribution in GBM requires spatial pharmacokinetic sampling, receptor-pathway profiling in human tissue, and validation in models that preserve regional BBB/BTB heterogeneity [6,7].

## 3. Albumin-Based Nanoparticle Formulations for GBM

### 3.1. Nanoparticle Albumin-Bound Paclitaxel, Nab-Paclitaxel, Abraxane^®^

Nanoparticle albumin-bound paclitaxel, commonly known as nab-paclitaxel or Abraxane^®^, remains the clinically most advanced albumin-based paclitaxel formulation and provides the main translational reference point for albumin-associated drug delivery in brain tumors. It consists of paclitaxel noncovalently associated with human serum albumin and forms a solvent-free, albumin-stabilized nanoparticle preparation with an approximate particle size of 130 nm [22,31]. This formulation addresses a central limitation of conventional solvent-based paclitaxel, namely the need for Cremophor EL and ethanol to compensate for poor aqueous solubility [32]. Because Cremophor EL has been associated with hypersensitivity reactions, altered pharmacokinetics, erythrocyte aggregation, abnormal lipoprotein patterns, and peripheral neurotoxicity, solvent avoidance represents a clinically relevant advantage of nab-paclitaxel [32].

From a formulation standpoint, eliminating Cremophor EL is a clinically practical benefit of nab-paclitaxel: patients can receive the drug without routine corticosteroid or antihistamine premedication, and the hypersensitivity burden that complicates conventional solvent-based schedules is substantially reduced [33]. Beyond this tolerability gain, the albumin matrix introduces a second, mechanistically distinct rationale—engaging endogenous albumin-handling pathways that may redirect the drug toward tumor vasculature and stroma. Desai et al. demonstrated that ABI-007 achieved greater endothelial transport and higher intratumoral paclitaxel concentrations than its Cremophor-based counterpart in preclinical models, suggesting that the albumin scaffold does more than dissolve a hydrophobic drug [22]. These transport effects, however, are context-dependent: receptor availability, vascular phenotype, and regional BBB/BTB integrity will all modulate the extent to which this biological advantage is realized in a given tumor compartment [12,22].

In GBM, paclitaxel potency is only one part of the problem; the larger translational challenge is achieving sufficient exposure across the BBB/BTB and within spatially heterogeneous tumor compartments. In patient-derived intracranial glioma xenografts, low-intensity pulsed ultrasound with microbubbles increased delivery of albumin-bound paclitaxel to brain and tumor regions and was associated with improved survival [34]. In the same study, Cremophor-based paclitaxel caused greater local neurotoxicity, including hemorrhage and necrosis, whereas ultrasound-assisted albumin-bound paclitaxel showed better tolerability under the tested conditions [34]. The key implication is that the therapeutic window of paclitaxel in GBM is shaped not only by the payload itself, but also by the formulation vehicle and the method used to modulate BBB/BTB access.

Clinical translation is supported by a phase I trial in recurrent GBM, in which an implantable nine-emitter ultrasound device was used to repeatedly open the BBB during intravenous administration of albumin-bound paclitaxel [14]. This study demonstrated the feasibility and pharmacokinetic enhancement of peritumoral brain delivery, providing early evidence of safety and delivery rather than definitive proof of survival benefit [14]. Nab-paclitaxel, therefore, represents a clinically established albumin-bound formulation that may be adapted to GBM delivery strategies if BBB/BTB opening, regional pharmacokinetics, neurotoxicity, and patient selection are rigorously evaluated.

### 3.2. Modified Albumin Nanoparticle Formulations

Recognizing that nab-paclitaxel was developed for systemic oncology rather than for brain tumor delivery, several groups have asked whether albumin-based nanocarriers can be engineered to improve BBB traversal beyond passive vascular leakage. The dual-drug nanoparticle platform reported by Lin et al. is among the more instructive attempts to answer this question [21]. By exploiting the hydrophobicity of paclitaxel and fenretinide to drive spontaneous albumin self-assembly—without crosslinkers that might compromise protein biocompatibility—they produced a nanoparticle that outperformed free drug in intracranial glioma models. The mechanistic explanation centered on SPARC and gp60 overexpression in glioma vasculature and stroma, which appeared to facilitate both transcytosis and local retention. What this system illustrates most clearly, however, is that albumin-mediated BBB delivery is an engineerable property, not a fixed one: the outcome depends critically on surface design, payload chemistry, and the receptor landscape of the specific tumor model being tested [12,21]. From a translational standpoint, the key implication of the Lin et al. platform is not that albumin alone ensures BBB penetration, but that albumin can serve as a tunable scaffold when payload chemistry, surface modification, and receptor biology are engineered together.

Low-molecular-weight protamine (LMWP) functionalization added a second design layer to this dual-drug albumin platform. Compared with unmodified controls, LMWP-modified albumin nanoparticles showed enhanced BBB penetration, intratumoral infiltration, and cellular uptake [21]. In subcutaneous and intracranial glioma models, the LMWP-functionalized paclitaxel/fenretinide nanoparticles elicited stronger therapeutic responses with reduced toxic side effects, accompanied by induction of apoptosis, antiangiogenic activity, and modulation of the tumor immune microenvironment [21]. These findings support albumin as a modular co-delivery scaffold, with the strongest conclusion applying to this specific engineered construct rather than to albumin formulations as a class.

Ligand-directed modification offers another route to increase cellular specificity. However, the GBM-specific evidence for albumin-based ligand systems is currently less developed than that for LMWP-modified albumin nanoparticles. Cyclic arginine–glycine–aspartic acid (cRGD) peptides target αvβ3/αvβ5 integrins, which are associated with tumor angiogenesis and glioma neovasculature, and have been widely explored in nanoparticle design for receptor-mediated uptake [5,27]. Because many cRGD-functionalized glioma studies rely on non-albumin polymeric or inorganic platforms, cRGD is best considered a transferable targeting concept rather than a fully validated albumin-specific GBM strategy.

Cell-mediated albumin-drug delivery extends the platform concept by using migratory immune cells as drug carriers. In a microfluidic GBM-on-a-chip system, macrophages loaded with nab-paclitaxel released approximately 40% of the payload within 24 h and about 80% by 48 h and showed greater anti-GBM activity than the free drug under the experimental conditions [29]. The same study also reported a shift toward an M1-like proinflammatory phenotype, suggesting that macrophage-based delivery can combine tumor tropism, controlled release, and immune-context interaction within a single experimental platform [29]. More broadly, cell-mediated nanoparticle delivery has been proposed as a complementary strategy for transporting therapeutic payloads across the BBB/BTB by exploiting the migratory and tumor-homing properties of engineered or drug-loaded cells [35]. For translation, the critical variables are carrier viability, migratory behavior, off-target release, immunotoxicity, phenotype stability, and function within brain-specific immune niches.

The paclitaxel/fenretinide co-loading strategy highlights an important pharmacologic issue in GBM: a single cytotoxic mechanism is unlikely to be sufficient for a biologically heterogeneous tumor. Building complementarity into the nanocarrier itself, rather than co-administering two separate formulations, has practical translational relevance [21,36]. Combining microtubule destabilization with retinoid-driven apoptosis and antiangiogenic signaling within one particle means that delivery of the carrier translates directly into delivery of both pharmacological hits. That said, co-loading is not without cost. Controlling the molar ratio of two structurally dissimilar drugs throughout manufacturing, storage, and in vivo release is technically demanding, and demonstrating that the ratio at the tumor site matches the ratio that produced synergy in vitro remains an unresolved requirement for clinical translation.

From a translational standpoint, albumin-based nanoparticle formulations for GBM fall into three categories: clinically established nab-paclitaxel, engineered albumin nanoparticles with peptide or ligand modifications, and biologically responsive or multi-payload systems. Nab-paclitaxel provides the strongest precedent for clinical formulations; LMWP-functionalized dual-drug albumin nanoparticles provide direct GBM-relevant preclinical evidence; and macrophage-loaded or ligand-directed systems remain exploratory strategies. Across all categories, albumin is best positioned as a versatile carrier scaffold rather than a universal targeting solution, with performance determined by payload chemistry, BBB/BTB context, receptor-pathway availability, tumor-region heterogeneity, and compatibility with adjunctive delivery technologies.

## 4. Mechanisms of Action and Pharmacodynamics

### 4.1. BBB/BTB Transport, Whole-Body Biodistribution, and Tumor Accumulation

Albumin-based carriers may improve brain-tumor delivery only when systemic biodistribution, endothelial transport, regional BBB/BTB permeability, tumor retention, and payload release are aligned within the same therapeutic context [5,10]. As summarized in Table 1, albumin-associated accumulation can arise from several mechanisms, including gp60/caveolin-mediated endothelial trafficking, SPARC-associated retention, FcRn-mediated systemic persistence, passive BTB leakage, and formulation-dependent uptake or release [12,19,20]. These mechanisms should not be interpreted as GBM-selective targeting pathways, because albumin handling also occurs in normal vascular and stromal compartments and can influence distribution outside the tumor [12,19,37].

Gp60/albondin is best understood as an endothelial albumin-binding pathway that can promote caveola-associated albumin transport rather than as a tumor-specific receptor [19,20]. This distinction matters for whole-body biodistribution because gp60/caveolin-mediated albumin handling may occur in non-tumor endothelial beds as well as tumor-associated vasculature, making regional vascular phenotype and BBB/BTB status critical determinants of carrier exposure [12,26]. In GBM, gp60-associated trafficking may contribute to endothelial crossing in selected vascular niches, but it cannot be assumed to overcome tight-junction restriction at BBB-preserved invasive margins [6,7]. SPARC provides a complementary but equally context-dependent retention mechanism. SPARC is an albumin-binding matricellular protein involved in cell–matrix interaction, extracellular matrix organization, collagen remodeling, growth-factor signaling, and vascular biology, and its effects vary by tissue context, tumor type, stromal state, and stage of disease [12,22,37,38]. In glioma models, albumin nanoparticles and albumin-binding photosensitizers have been linked to SPARC-associated uptake or retention, including reduced uptake after SPARC knockout in a glioma photosensitizer model [21,23]. However, SPARC expression in normal matrix remodeling, stromal remodeling, inflammatory or repair-associated tissue states, and multiple cancer contexts limits its interpretation as a GBM-specific selectivity marker [24,37,38]. Consistent with this caution, SPARC expression did not predict the efficacy of nab-paclitaxel plus gemcitabine or gemcitabine alone in the exploratory biomarker analysis of the phase III MPACT in metastatic pancreatic cancer [24].

The translational challenge in GBM is therefore not simply whether gp60 or SPARC is present, but where, in which cell type, and under what barrier state these pathways are active. Receptor expression, vascular permeability, stromal composition, and immune context may differ between the enhancing tumor core, perivascular regions, necrotic or hypoxic compartments, and BBB-preserved infiltrative margins [6,7]. Treatment can further reshape vascular and stromal states, making post-treatment and recurrent GBM particularly important settings for validating albumin-associated transport mechanisms [39]. Future studies should therefore quantify tumor-to-normal brain exposure, invasive-margin penetration, receptor-positive niche localization, and off-target normal-tissue distribution rather than relying on bulk tumor accumulation alone [7,10]. Mechanistic attribution should be supported by spatial pharmacokinetics, immunohistochemistry, receptor-blocking or knockdown experiments, single-cell or spatial transcriptomic profiling, and model-to-patient tissue matching [10,21,23].

### 4.2. Cytotoxic Mechanisms

Paclitaxel stabilizes polymerized microtubules, disrupts mitotic progression, and can trigger mitotic arrest and apoptosis, making it pharmacologically attractive for proliferative tumor compartments [40]. In GBM, however, intrinsic cytotoxic potency does not necessarily translate into intracranial efficacy because therapeutically meaningful exposure must be achieved across regionally heterogeneous BBB/BTB compartments, efflux-transporter barriers, and protected invasive margins [5,7].

Albumin-based formulation may improve the therapeutic behavior of paclitaxel by increasing its aqueous compatibility, modifying systemic exposure, and enabling integration with BBB/BTB-opening strategies [14,22]. These effects should be interpreted as delivery- and formulation-dependent modulation of payload exposure rather than as a change in the core cytotoxic mechanism of paclitaxel [10,40]. Accordingly, cytotoxic efficacy in GBM should be evaluated by linking formulation design to spatial pharmacokinetics, tumor-cell exposure, invasive-margin penetration, and local neurotoxicity rather than by in vitro potency alone [6,7].

### 4.3. Immunomodulatory Effects

Paclitaxel’s interaction with the immune microenvironment adds a layer of pharmacological complexity that is often underappreciated in the GBM delivery literature. In peripheral tumor models, the drug can repolarize tumor-associated macrophages toward a pro-inflammatory phenotype via Toll-like receptor 4 (TLR4)-dependent signaling [41], and the albumin-bound formulation itself appears to augment myeloid engagement—macropinocytosis of nab-paclitaxel by macrophages triggered immunostimulatory cytokine release in pancreatic cancer experiments [28]. Whether analogous effects occur in the glioblastoma immune microenvironment, which is dominated by brain-resident microglia and highly tolerogenic myeloid populations rather than the macrophage subtypes studied in pancreatic cancer, is far from established.

A more deliberate attempt to harness macrophage repolarization was reported by Vadevoo et al., who conjugated interleukin-4 (IL-4) receptor-targeting peptides to nab-paclitaxel with the explicit goal of redirecting immunosuppressive M2-like macrophages toward an anti-tumor M1-like state via reactive oxygen species (ROS)-high-mobility group box 1 (HMGB1)-TLR4 signaling [42]. This strategy is mechanistically interesting, but its interpretation in GBM requires caution: the myeloid landscape of brain tumors, in which microglia and bone marrow-derived macrophages coexist in region-specific proportions shaped by the blood-brain barrier itself, is qualitatively different from the peripheral tumor models in which this strategy was evaluated.

Together, these non-GBM studies suggest that albumin-bound paclitaxel can interact actively with myeloid compartments through macrophage uptake, cytokine induction, and phenotype modulation; however, these findings should be interpreted as immune-context precedent rather than direct evidence that albumin-based formulations reprogram the GBM immune microenvironment.

Accordingly, future studies should determine whether albumin-based carriers alter macrophage/microglial uptake, T-cell infiltration, cytokine signaling, antigen presentation, and immune-checkpoint responsiveness in immunocompetent, humanized, or tumor–immune co-culture models. These questions become especially important when albumin-based delivery is combined with immune checkpoint blockade, chimeric antigen receptor (CAR) T cells, radiotherapy, or metabolic interventions aimed at reversing the immunosuppressive GBM microenvironment.

## 5. Preclinical Evidence and In Vivo Efficacy

### 5.1. Orthotopic Glioma Models

Orthotopic xenograft systems and genetically engineered mouse models (GEMMs) are particularly useful for evaluating albumin-based drug delivery in glioblastoma because they better capture intracranial growth, tumor-brain interactions, vascular constraints, and, in selected cases, genotype-driven tumor evolution than conventional subcutaneous models [34,43,44]. In these systems, albumin-based or albumin-compatible paclitaxel delivery strategies have shown meaningful antitumor activity when paired with formulation engineering or barrier-modulating approaches. However, the findings are most informative when interpreted in relation to the vascular phenotype, BBB/BTB status, and spatial constraints of each model [34,43].

In patient-derived intracranial glioma xenografts, low-intensity pulsed ultrasound with microbubbles increased brain and tumor delivery of albumin-bound paclitaxel and prolonged survival, supporting the rationale for pairing albumin-bound chemotherapy with transient BBB/BTB modulation [34]. The same study reported greater local neurotoxicity with Cremophor-based paclitaxel, including hemorrhage and necrosis, whereas ultrasound-assisted albumin-bound paclitaxel showed better tolerability under the tested conditions. These findings indicate that the therapeutic window of paclitaxel in GBM is shaped by formulation chemistry, BBB/BTB-opening modality, regional drug exposure, and local brain tolerability rather than by payload potency alone [14,34].

Albumin nanoparticles co-encapsulating paclitaxel and fenretinide demonstrate how albumin-based systems can be engineered for multi-payload delivery. Lin et al. generated dual-drug-loaded albumin nanoparticles through albumin self-assembly, and low-molecular-weight protamine functionalization enhanced BBB penetration, intratumoral infiltration, and cellular uptake in glioma models [21]. These nanoparticles improved therapeutic outcomes in both subcutaneous and intracranial glioma models, with the strongest conclusion being validation of this specific engineered formulation rather than general proof that all albumin formulations achieve equivalent BBB penetration [21].

Optical BBB/BTB modulation studies add an important spatial dimension by showing that delivery efficacy depends on tumor phenotype and regional vascular biology. Although not albumin-specific, these studies are relevant to albumin-compatible delivery because they illustrate how regional barrier modulation can alter paclitaxel access in spatially distinct glioblastoma compartments. In genetically engineered glioblastoma models, optical BBB/BTB modulation using vascular-targeted gold nanoparticles and pulsed laser excitation reversibly increased barrier permeability and enhanced paclitaxel delivery across angiogenic-core and infiltrative-margin phenotypes [43]. This approach reduced tumor burden and prolonged survival, with the therapeutic effect being stronger in infiltrative tumors, where drug access is typically constrained by preserved vascular barriers [43]. These findings shift the preclinical question from whether tumor burden is reduced to where, how deeply, and under what vascular conditions the payload reaches tumor-infiltrated brain, making spatial pharmacokinetics, BBB/BTB integrity, receptor-pathway validation, and toxicity essential endpoints for albumin-compatible delivery [6,10,12].

### 5.2. Combination Strategies, Albumin-Binding Payloads, and Drug Interactions

The most pharmacologically grounded combination for albumin-bound paclitaxel in GBM is its pairing with the standard-of-care alkylating agent temozolomide. Qu et al. reported that nab-paclitaxel increased temozolomide sensitivity in GBM cells by disrupting DNA-damage repair and promoting ferroptosis-related vulnerability [45]. This finding supports a payload-specific combination rationale, but it should not be overextended as evidence that albumin delivery itself overcomes temozolomide resistance [45]. For albumin-based GBM delivery, the translational question is whether improved formulation and BBB/BTB access can deliver this pharmacological interaction to relevant tumor compartments without unacceptable systemic or CNS toxicity [7,14]. Non-albumin local-delivery studies, such as paclitaxel–temozolomide co-loaded hydrogel systems, support taxane–alkylator compatibility but should be interpreted as pharmacologic rather than albumin-specific delivery evidence [46]. What the albumin-bound formulation may add to this pharmacological rationale is the possibility of coordinating systemic paclitaxel exposure with transient BBB/BTB access, a delivery dimension that differs from both local hydrogel delivery and conventional systemic free-drug administration [14,34].

A direct preclinical test of SPARC-associated albumin retention is provided by the photodynamic therapy study by Li et al. [23]. Their albumin-binding zinc phthalocyanine photosensitizer achieved approximately 83.6% tumor growth inhibition and a 14-day median survival extension in a glioma model; when SPARC was knocked out, photosensitizer uptake fell by 63.1% [23]. This knockdown experiment is methodologically important because it supports a functional, not merely associative, contribution of SPARC to albumin-carrier accumulation in this specific system [23]. Beyond the photodynamic therapy application itself, the study supports the use of albumin-binding design for light-activatable or theranostic payloads in SPARC-expressing glioma contexts [23].

Taken together, these studies suggest that albumin-based delivery is most preclinically informative when it addresses a defined pharmacologic problem, such as poor solubility, inadequate regional exposure, insufficient tumor retention, or coordination of combination payloads. Their preclinical value is strongest when formulation design is linked to measurable delivery endpoints, including brain/tumor pharmacokinetics, invasive-margin penetration, receptor-associated uptake, or treatment-site toxicity [10,12]. Future experiments should therefore be judged by the linkage among mechanism, exposure, and efficacy rather than by tumor volume reduction alone [6,10].

## 6. Clinical Translation and Current Clinical Evidence

### 6.1. Clinical Trial Landscape of Albumin-Based Therapeutics in Brain Tumors

Despite the deep extracranial clinical experience with albumin-bound drug products, GBM-directed clinical translation remains limited, and the field is still largely in a proof-of-concept stage. The most directly informative published human data come from the phase I component of NCT04528680, a recurrent glioblastoma study in which an implanted nine-emitter ultrasound device was used to open the BBB repeatedly prior to intravenous nab-paclitaxel administration at escalating doses from 40 to 260 mg/m^2^ [14]. A key strength of this study is that intraoperative specimens obtained after sonication and immediate drug administration allowed direct quantification of tissue drug concentrations, making the trial particularly informative for spatially resolved CNS drug exposure in recurrent GBM [14]. The regimen was feasible, although dose-related neurologic toxicity occurred, including reversible grade 3 taxane-associated encephalopathy at the 260 mg/m^2^ dose level, which was classified as dose-limiting toxicity [14].

The extracranial success of nab-paclitaxel supports formulation maturity, dosing experience, and systemic safety characterization, but it should not be interpreted as evidence that gp60- or SPARC-mediated uptake is the dominant determinant of efficacy in GBM [10,24,33]. Clinical translation should therefore distinguish formulation precedent, CNS pharmacokinetic proof of concept, and therapeutic efficacy validation [14]. For clarity, we distinguish five evidence levels across albumin-based and albumin-compatible strategies: clinical efficacy evidence in extracranial cancers, clinical pharmacokinetic evidence in GBM, direct orthotopic GBM preclinical evidence, mechanistic or biomarker evidence without therapeutic validation, and exploratory or speculative approaches requiring further validation. To avoid conflating extracranial formulation success with GBM-specific therapeutic efficacy, the studies summarized in Table 2 are categorized by their principal role in the evidence. Extracranial phase III trials are included as precedents for formulation, safety, and systemic combination of albumin-bound paclitaxel, whereas the recurrent GBM low-intensity pulsed ultrasound with microbubbles (LIPU/MB) study provides direct CNS pharmacokinetic proof of concept rather than definitive therapeutic validation. The pancreatic cancer MPACT biomarker analysis is included to highlight that SPARC should not be regarded as a universal predictive biomarker for albumin-bound therapeutics. Nab-sirolimus is presented as exploratory brain tumor evidence that extends albumin-bound drug development beyond taxane delivery, but mature GBM-specific efficacy conclusions remain limited.

Beyond albumin-bound paclitaxel, nab-sirolimus, also known as ABI-009, extends albumin-bound therapeutic development toward pathway-directed mechanistic target of rapamycin (mTOR) inhibition. NCT03463265 evaluated nab-sirolimus in recurrent high-grade glioma and newly diagnosed glioblastoma as an open-label phase II study, either as monotherapy or in combination with temozolomide, bevacizumab, lomustine, marizomib, or radiotherapy [53]. In addition, a pediatric phase I study evaluated nab-sirolimus with temozolomide and irinotecan in recurrent or refractory solid tumors, including CNS tumors, and established dose-limiting toxicities, pharmacokinetics, and a recommended dosing framework for this combination strategy [54]. Because this phase I study enrolled pediatric patients with recurrent or refractory solid tumors, including CNS tumors, its main relevance to GBM translation is to provide combination safety, pharmacokinetic, and dosing precedent for nab-sirolimus rather than disease-specific efficacy evidence. Together, these studies broaden the clinical scope of albumin-bound therapeutics beyond taxane delivery, although mature GBM-specific efficacy conclusions remain limited.

Taken together, available clinical studies show that albumin-based therapeutics have substantial clinical precedent in extracranial solid tumors and are beginning to enter brain tumor-specific clinical testing. The extracranial trials support formulation maturity, dosing experience, characterization of systemic safety, and the feasibility of combination chemotherapy, but they do not demonstrate that albumin-receptor pathways are sufficient to drive clinical efficacy in GBM. The current evidence on brain tumors remains early-stage and primarily supports feasibility, safety assessment, pharmacokinetic enhancement, and combination rationale rather than a definitive survival benefit. The LIPU/MB-albumin-bound paclitaxel trial provides the most direct GBM-specific proof of concept to date. In contrast, nab-sirolimus illustrates the extension of albumin-bound platforms beyond taxane delivery to pathway-directed therapeutic strategies.

ANG1005, a non-albumin Angiopep-2–paclitaxel conjugate designed to exploit low-density lipoprotein receptor-related protein 1 (LRP1)-mediated BBB transcytosis, provides a useful cautionary comparator for CNS drug delivery. In a phase II trial in recurrent high-grade glioma, ANG1005 did not meet prespecified efficacy endpoints despite an acceptable taxane-class safety profile [55]. This result underscores that enhanced BBB transport must be coupled with tumor retention, spatial penetration, tumor-cell susceptibility, and biomarker-guided stratification to translate into meaningful clinical benefit [55].

We therefore interpret the current clinical evidence as delivery-oriented proof of concept rather than therapeutic validation. Importantly, increased peritumoral drug exposure should be interpreted as a delivery endpoint, not as a surrogate for durable tumor control, invasive-margin eradication, or survival benefit. Albumin-bound paclitaxel, combined with BBB opening, has demonstrated feasibility, regional CNS pharmacokinetic enhancement, and a manageable dose-escalation experience. However, randomized evidence of survival benefit, durable radiographic response, neurocognitive preservation, and quality-of-life improvement is not yet available. Future GBM trials should explicitly separate delivery success from therapeutic success by pairing spatial pharmacokinetics with survival, neurocognitive, quality-of-life, and biomarker-enriched endpoints. This distinction is essential because increased peritumoral exposure does not necessarily guarantee eradication of the invasive margin or durable disease control.

### 6.2. Safety and Tolerability

Nab-paclitaxel offers a practical tolerability advantage over conventional Cremophor EL-containing paclitaxel by avoiding solvent-associated hypersensitivity concerns and reducing the need for routine corticosteroid and antihistamine premedication [33]. This advantage of solvent-free formulation is clinically meaningful, but it does not eliminate taxane-associated toxicity. Peripheral neuropathy remains one of the most clinically relevant dose-limiting toxicities of nab-paclitaxel; a meta-analysis of prospective phase II/III trials reported all-grade peripheral neuropathy in 51.0% of nab-paclitaxel-treated patients and high-grade neuropathy in 12.4% [56]. A subsequent systematic review and meta-analysis comparing nab-paclitaxel with solvent-based paclitaxel suggested a higher incidence of peripheral neuropathy with nab-paclitaxel in mono-chemotherapy settings. However, dosage, schedule, disease context, and assessment method influence cross-trial comparisons [57]. Accordingly, nab-paclitaxel is best described as solvent-free and clinically manageable, not as uniformly less neurotoxic than solvent-based paclitaxel.

Myelosuppression is another clinically important toxicity requiring careful dose monitoring. Population pharmacokinetic–pharmacodynamic modeling in Japanese patients with metastatic solid tumors showed that nab-paclitaxel-associated neutropenia is exposure-dependent and that age and serum albumin levels may influence neutrophil count dynamics [58]. These findings favor individualized dose adjustment, hematologic monitoring, and selective supportive care over fixed safety assumptions across patient populations. In the GBM setting, systemic taxane toxicities must be considered alongside neurologic safety because BBB-opening strategies may increase CNS exposure while preserving systemic risks.

Although early clinical experience combining albumin-bound paclitaxel with LIPU/MB-mediated BBB opening supports the feasibility of repeated sonication-assisted delivery, larger clinical datasets are required to define neurotoxicity, cumulative neuropathy risk, hematologic tolerability, and long-term safety [14]. The occurrence of reversible taxane-associated encephalopathy at the highest tested dose level supports careful dose escalation, imaging-based monitoring, neurologic assessment, and pharmacokinetic sampling in future GBM trials [14]. The current clinical evidence, therefore, supports feasibility and pharmacokinetic enhancement. At the same time, definitive safety optimization and efficacy validation are still needed before albumin-bound paclitaxel can be established as a GBM-directed therapeutic strategy.

Safety considerations for non-taxane albumin-based therapeutics need to be assessed separately according to payload biology. Nab-sirolimus introduces mTOR inhibitor-associated toxicities rather than taxane-associated neuropathy or myelosuppression, and its brain tumor trial design includes both monotherapy and combination regimens with standard or investigational agents [53]. As albumin-based platforms diversify beyond paclitaxel, safety assessment should distinguish formulation-related risks from payload-specific toxicity, CNS exposure, combination-partner effects, and BBB-modulation-related neurologic risk.

## 7. Mechanistic and Translational Advantages of Albumin-Based Delivery for GBM

### 7.1. Mechanistic Advantages Beyond Passive Accumulation

The enhanced permeability and retention (EPR) effect, which underlies many passive nanomedicine delivery rationales, is particularly unreliable in GBM because vascular permeability is spatially heterogeneous and often does not extend to infiltrative tumor margins [6,7,25]. Although contrast-enhancing tumor regions may show disrupted BTB permeability, recurrent disease commonly arises from tumor-infiltrated brain regions where BBB integrity is relatively preserved, limiting the value of passive accumulation alone [5,59]. The potential advantage of albumin-based platforms is that they may combine formulation-level benefits with biologically interpretable transport and retention mechanisms. Albumin-associated systems can improve solubilization of hydrophobic payloads and prolong systemic exposure, in part through albumin-binding capacity and FcRn-mediated recycling [10,11,17]. They may also engage albumin-handling pathways such as gp60/caveolin-associated endothelial transport and SPARC-associated retention under selected biological contexts [12,22]. These mechanisms should be interpreted as candidate contributors rather than validated GBM-selective targeting pathways. Their translational value depends on whether they increase drug exposure in clinically relevant compartments, including peritumoral brain and invasive margins, without increasing unacceptable normal-brain or systemic toxicity [6,14].

### 7.2. Platform Modularity and Combination Compatibility

A second advantage of albumin-based delivery is platform modularity. Albumin can serve as a solubilizing matrix, a nanoparticle-forming scaffold, an albumin-binding partner, or a functionalizable carrier for structurally diverse payloads, including cytotoxics, pathway-directed agents, photosensitizers, and potentially imaging or theranostic constructs [8,10,23]. This modularity is relevant to GBM because effective therapy will likely require combinations that coordinate payload activity with BBB/BTB access, regional tumor exposure, and local tolerability rather than simply increasing systemic dose intensity.

The evidence reviewed above illustrates this principle across preclinical and early clinical contexts. Nab-paclitaxel–temozolomide studies support a payload-specific combination rationale, albumin-binding photosensitizers support light-activatable or theranostic design in selected SPARC-expressing glioma contexts, and nab-sirolimus extends albumin-bound development toward pathway-directed mTOR inhibition. These examples should not be interpreted as interchangeable evidence for all albumin formulations. Instead, they show that albumin-based delivery is most informative when formulation design is matched to a defined pharmacological problem, such as poor solubility, inadequate regional exposure, insufficient tumor retention, or the need to coordinate systemic therapy with BBB/BTB-opening technologies.

### 7.3. Translational Positioning

The principal translational advantage of albumin-based delivery is not that albumin guarantees tumor selectivity, but that clinically familiar albumin-bound chemistry can be integrated with spatial pharmacokinetic testing, BBB/BTB-opening strategies, and biomarker-informed trial design. This positioning differentiates albumin-based systems from purely experimental nanocarriers while still requiring disease-specific validation in GBM. The available evidence supports formulation maturity, systemic dosing experience, and early CNS pharmacokinetic proof of concept, but it does not yet establish durable therapeutic efficacy in GBM [10,14].

Accordingly, the translational value of albumin-based delivery should be judged by whether it improves clinically meaningful exposure at protected tumor sites, preserves normal-brain safety, enables rational combinations, and produces radiographic, survival, neurocognitive, or quality-of-life benefit in appropriately selected patients. This framing positions albumin-based delivery as a promising but still unproven strategy whose future development should be guided by spatial pharmacokinetics, receptor-pathway validation, BBB/BTB heterogeneity, and prospective go/no-go criteria [6,10,14]. To make this translational framework actionable, albumin-based GBM delivery platforms should be evaluated using predefined go/no-go decision domains rather than by formulation novelty or tumor-volume reduction alone. Table 3 summarizes a concise framework for judging whether an albumin-based platform should advance from formulation optimization to early clinical testing or from delivery proof of concept to therapeutic efficacy evaluation.

This framework separates delivery feasibility from therapeutic validation. In early development, advancement may be justified when a formulation is reproducible, increases CNS or peritumoral exposure, and maintains an acceptable safety profile. However, later-stage development should require evidence that increased exposure reaches clinically relevant tumor compartments, particularly BBB-preserved invasive margins, and produces payload-specific pharmacodynamic activity or patient-centered benefit. This distinction is important because albumin-associated uptake pathways, including gp60/caveolin-associated transport, FcRn-mediated recycling, and SPARC-associated retention, may contribute to biodistribution but do not by themselves establish GBM selectivity or therapeutic efficacy [10,12,14,24]. Therefore, albumin-based platforms should progress clinically only when spatial pharmacokinetics, mechanistic validation, safety, and efficacy endpoints are aligned.

## 8. Limitations and Challenges

### 8.1. Spatially Heterogeneous Tumor Penetration and Invasive-Margin Delivery

Even when albumin-based formulations enhance BBB/BTB transport in selected biological contexts, glioblastoma remains difficult to treat because drug delivery is spatially heterogeneous. Passive nanoparticle accumulation is intrinsically unreliable in human solid tumors, and the problem is amplified in GBM because BBB/BTB permeability varies substantially across the enhancing tumor core, perivascular regions, and infiltrative margins [25,59]. The tumor core may contain regions of abnormal angiogenesis and barrier disruption, whereas invasive margins often retain more intact BBB features, making them less accessible to systemically administered agents [6].

For albumin-bound drugs, this spatial barrier is clinically relevant because improved delivery to peritumoral or barrier-disrupted regions can still leave other tumor compartments underexposed. The phase 1 clinical experience with low-intensity pulsed ultrasound and microbubbles demonstrated that BBB opening can increase peritumoral exposure of albumin-bound paclitaxel in recurrent GBM, while also highlighting the need to measure regional pharmacokinetics rather than assume homogeneous drug distribution [14]. At the invasive edge, subtherapeutic exposure may allow residual tumor cells to persist, contributing to local recurrence and therapeutic escape [6]. Consequently, future studies need to report not only bulk tumor accumulation but also invasive-margin delivery, perivascular distribution, tumor-to-normal brain ratios, and spatially resolved payload retention.

### 8.2. Molecular Resistance and Biomarker Uncertainty

Resistance to albumin-bound paclitaxel in GBM is expected to reflect both tumor-intrinsic drug-response programs and delivery-associated barriers. Genome-scale clustered regularly interspaced short palindromic repeats (CRISPR) screening and analyses of an orthotopic glioma model identified the translocon-associated protein subunit SSR3, signal sequence receptor subunit 3, as a candidate predictor of paclitaxel susceptibility, and SSR3 expression correlated with taxane response across glioma and breast cancer systems [60]. This finding is important, but SSR3 remains a putative biomarker and requires prospective validation before it can guide patient selection in GBM.

The unfolded protein response may also influence treatment adaptation, although its relationship to paclitaxel response remains insufficiently defined. A brain-permeable inositol-requiring enzyme (IRE) 1 kinase inhibitor has shown activity as an adjuvant strategy with temozolomide in preclinical GBM models, positioning IRE1α as an exploratory stress-adaptation and resistance-associated target rather than a validated paclitaxel-specific biomarker [61]. Efflux transporters further complicate albumin-bound drug development because P-glycoprotein and related BBB transporters can limit CNS drug exposure and may also reduce intracellular drug accumulation after payload release [5]. Albumin-based formulation may improve solubility, systemic exposure, and tumor retention, but these advantages do not fully bypass cellular drug resistance, efflux-mediated exclusion, or adaptive survival programs. Payload-specific interactions, such as nab-paclitaxel–temozolomide sensitization, should therefore be interpreted as mechanistic hypotheses requiring biomarker-defined validation rather than as general evidence that albumin delivery overcomes resistance [45]. Rational clinical development needs to distinguish delivery failure, intracellular drug resistance, and payload-specific susceptibility.

### 8.3. Immunosuppressive and Metabolically Reprogrammed Tumor Microenvironment

Even if albumin-based delivery achieves adequate tumor exposure, the immunosuppressive architecture of GBM can independently negate therapeutic benefit. This is not merely an obstacle to immunotherapy; it also matters for chemotherapy, because immune-mediated survival signaling, stromal sequestration, and macrophage-driven metabolic reprogramming can reduce effective intracellular drug exposure independent of delivery success. The GBM immune landscape—encompassing brain-resident microglia, infiltrating myeloid cells, dysfunctional T-cell populations, and astrocyte-orchestrated suppression—is too complex and spatially heterogeneous to be described adequately by the M1/M2 dichotomy that dominates much of the nab-paclitaxel immunomodulation literature [30]. Myeloid phenotypes shift with tumor region, treatment history, and metabolic context, meaning that immunomodulatory claims derived from a single experimental time point or tumor model are likely incomplete.

Albumin-bound paclitaxel has been reported to promote macrophage activation in pancreatic cancer models, but its capacity to reprogram the GBM immune microenvironment remains uncertain [28]. Recent evidence that glioblastoma-instructed astrocytes can suppress tumor-specific T-cell immunity through an interleukin-11 (IL-11)-signal transducer and activator of transcription 3 (STAT3)-TNF-related apoptosis-inducing ligand (TRAIL) axis further supports the need for GBM-specific validation [62].

Metabolic reprogramming adds another barrier to effective albumin-based cytotoxic delivery. Recent evidence suggests that macrophage-derived lactate can reinforce glioblastoma stem-like cell survival and immunosuppression through lactate transport, DNA-repair modulation, and suppression of the cyclic GMP–AMP synthase (cGAS)–stimulator of interferon genes (STING) signaling [63]. The implication for albumin-based delivery is that drug access alone may be insufficient unless immune exclusion, astrocyte-mediated suppression, and metabolic immunosuppression are also addressed.

### 8.4. CMC, Reproducibility, and Scalability Constraints

Manufacturing complexity is frequently treated as a downstream problem in academic nanomedicine, but for albumin-based GBM candidates, it is a major determinant of clinical readiness. Albumin’s conformational sensitivity means that desolvation conditions, cross-linking chemistry, drying parameters, and protein-stabilization protocols each leave fingerprints on particle size distribution, drug-loading efficiency, and release kinetics—and small deviations in any of these parameters can propagate into meaningful differences in BBB interaction and intratumoral behavior [8,10]. Residual solvents, endotoxin content, and batch-to-batch variability are not peripheral quality concerns to be resolved late in development; they are the attributes on which regulatory reviewers will focus, and failure to define and control them early typically results in costly and time-consuming reformulation cycles before any GBM-specific clinical investigation can begin [13].

This issue is especially important for GBM applications because small changes in particle size, surface properties, albumin structure, or drug-release behavior may alter BBB/BTB interaction, systemic pharmacokinetics, immune recognition, and intratumoral penetration. Although albumin-bound paclitaxel provides an established manufacturing precedent, next-generation albumin nanoparticles, conjugates, or multifunctional constructs cannot automatically inherit the same chemistry, manufacturing, and controls profile [10]. Therefore, advanced albumin-based GBM formulations need early CMC planning, validated analytical assays, scale-up comparability, and good manufacturing practice (GMP)-compatible manufacturing before they can reliably progress to clinical testing [13].

### 8.5. Limited Disease-Specific Clinical Evidence in GBM

Substantial preclinical evidence exists for albumin-bound therapeutics, but mature disease-specific efficacy data in glioblastoma remain limited. The current standard treatment backbone for newly diagnosed GBM—maximal safe resection followed by radiotherapy with concomitant and adjuvant temozolomide—was established by the Stupp regimen, yet recurrence remains common and effective therapeutic options after relapse remain limited [2].

Albumin-based therapeutics are now entering brain tumor-specific clinical testing, but the available evidence remains in its early stages. The most direct GBM-specific clinical evidence comes from studies combining albumin-bound paclitaxel with ultrasound-mediated BBB opening, which primarily establish feasibility, safety, pharmacokinetic enhancement, and regional delivery to peritumoral brain tissue, not definitive survival benefit [14]. The broader clinical landscape also includes nab-sirolimus, also known as ABI-009 or nab-rapamycin, which has been evaluated in recurrent high-grade glioma and newly diagnosed GBM as an albumin-bound mTOR inhibitor. However, this evidence remains insufficient to establish clinical efficacy in GBM [53].

Evidence of increased peritumoral drug exposure or clinical feasibility does not, by itself, demonstrate durable antitumor efficacy, optimal patient selection, or superiority over existing salvage approaches. Randomized trials with clinically meaningful endpoints—including overall survival, progression-free survival, neurocognitive safety, corticosteroid requirement, quality of life, and radiographic response durability—are needed to define the therapeutic value of albumin-based delivery in newly diagnosed and recurrent GBM. Until such data mature, albumin-based approaches remain clinically promising and mechanistically rational, but not yet clinically proven for GBM.

### 8.6. Limitations of Preclinical Models

Many of the translational uncertainties outlined in this section trace back to a more fundamental problem: the preclinical models used to generate the supporting evidence are poorly matched to the biological situation that actually matters in GBM patients. Standard two-dimensional cultures strip away the BBB, the brain-specific extracellular matrix, vascular heterogeneity, and the immune context that collectively govern therapeutic response in vivo [64]. Subcutaneous xenograft models add an in vivo dimension but replace the intracranial anatomy with a peripheral growth environment that lacks the diffuse invasion pattern, spatially variable barrier integrity, and perivascular immune niches that define human GBM [6]. For albumin-based delivery specifically, this matters more than for conventional small-molecule drugs: the proposed advantages—SPARC-mediated retention, gp60-dependent transcytosis, BBB traversal—are context-dependent phenomena that may not occur in a subcutaneous tumor lacking glioma-typical vasculature. These limitations are particularly important for albumin-based formulations because therapeutic performance depends not only on cytotoxic potency but also on BBB/BTB transport, vascular accessibility, tumor retention, brain distribution, and penetration into protected tumor regions [10].

Preclinical development should place greater emphasis on models that closely mimic the human glioblastoma tumor microenvironment. In particular, next-generation GBM models need to incorporate patient-derived tumor heterogeneity, BBB/BTB physiology, spatially resolved invasive-margin biology, human immune context, therapy-driven ecosystem evolution, and albumin-specific transport mechanisms, including SPARC-, gp60/albondin-, FcRn-, and caveolin-associated pathways [12,64,65]. Representative model platforms and core evaluation endpoints for improving the translational relevance of albumin-based drug delivery systems in GBM are summarized in Table 4, with an expanded model-by-endpoint framework provided in Supplementary Table S1.

This table should be interpreted as a model-selection framework rather than a hierarchy of experimental systems. No single platform can fully recapitulate human GBM biology; therefore, model selection should be matched to the primary translational question, such as BBB/BTB transport, invasive margin penetration, albumin receptor dependency, immune cell interactions, recurrence biology, or compatibility with BBB/BTB-opening technologies. For albumin-based therapeutics, evaluation needs to move beyond bulk tumor volume reduction or survival extension and incorporate mechanism-informed delivery endpoints, including tumor-to-normal brain accumulation, BBB/BTB permeability, perivascular distribution, hypoxic core delivery, immune cell uptake, receptor-positive niche localization, and spatial concordance between the model and human GBM tissue [6,12].

Albumin-associated transport and retention require validation using profiling of gp60/albondin, SPARC, FcRn, and caveolin-1, supported, where possible, by immunohistochemistry, receptor-blocking assays, gene knockdown, single-cell RNA sequencing, or spatial transcriptomics [10,12]. This validation is important because apparent tumor accumulation may reflect nonspecific vascular leakiness, disrupted barrier integrity, or enhanced permeability and retention rather than true albumin-mediated transport [12]. This distinction matters because the clinical success of albumin-bound formulations in extracranial tumors does not establish gp60, SPARC, FcRn, or caveolin-associated pathways as universal predictors of response in GBM.

The immune context is another area where model selection is critical. Immunodeficient xenografts incompletely capture interactions among albumin carriers, tumor-associated macrophages, microglia, astrocytes, lymphocytes, and myeloid-derived suppressor cells [64]. This limitation is clinically relevant because glioblastoma-instructed astrocytes can suppress tumor-specific T-cell immunity through an IL-11-STAT3-TRAIL axis, which supports the use of models that preserve brain-resident stromal and immune compartments [62]. Accordingly, immunocompetent syngeneic models, humanized orthotopic systems, and autologous tumor–immune organoid co-cultures are most appropriate when immune-mediated efficacy, immunotoxicity, macrophage/microglial uptake, or immunotherapy combinations are central to the study question.

Preclinical systems also need to reflect treatment history and disease evolution. Most patients receive maximal safe resection, radiotherapy, and temozolomide before recurrence, and single-cell analyses of matched primary and recurrent glioblastomas show that therapy can reshape malignant cell states and nonmalignant ecosystem components [2,39]. Post-treatment and recurrence-mimicking models can help determine how therapy-induced vascular remodeling, immune reprogramming, altered albumin-receptor expression, and BBB/BTB changes affect nanocarrier distribution and therapeutic response [39].

## 9. Future Perspectives and Optimization Strategies

Future development of albumin-based drug delivery systems for glioblastoma needs a transition from empirical formulation testing to mechanism-guided, biomarker-informed, and clinically synchronized strategies. The most practical priorities are rational integration with BBB/BTB-opening technologies, molecular selection of patients most likely to benefit from albumin-mediated transport, biologically justified combination regimens, advanced multifunctional formulation design, and scalable manufacturing platforms [10,13].

A practical development path would be a staged translational workflow rather than parallel empirical testing: first, confirm albumin-carrier biodistribution and invasive-margin penetration using spatial pharmacokinetic assays; second, validate albumin-associated transport or retention pathways, including SPARC, gp60/albondin, FcRn, and caveolin-1, in model systems matched to human GBM tissue; third, define payload-specific sensitivity and resistance biomarkers such as SSR3, efflux transporter activity, DNA-damage repair status, or ferroptosis-associated vulnerability; fourth, synchronize drug administration with BBB/BTB-opening parameters; and finally, test clinically meaningful endpoints including survival, neurocognitive safety, corticosteroid dependence, quality of life, and durable radiographic control.

### 9.1. Rational Combination with BBB-Opening Technologies

Among the strategies discussed in this section, coupling albumin-bound paclitaxel with reversible BBB opening via focused ultrasound and microbubbles currently has the most direct GBM-specific pharmacokinetic proof of concept for albumin-based delivery. The phase I component of NCT04528680 already demonstrated in humans that LIPU/MB-mediated opening with an implanted nine-emitter device measurably increased peritumoral paclitaxel concentrations. This finding is pharmacokinetically meaningful rather than merely logistically feasible [14]. The broader feasibility of repeated BBB-opening procedures in patients with high-grade glioma is supported by the multicenter BT008NA trial, in which microbubble-enhanced transcranial focused ultrasound was combined with temozolomide [67]. Magnetic resonance (MR)-guided focused ultrasound extends the technological repertoire by enabling non-invasive, spatially targeted BBB opening guided by real-time imaging. However, workflow standardization—including reproducibility of coverage, imaging quality thresholds, and patient tolerability—must be established before this approach can be reliably integrated into neuro-oncology practice [68]. Optical BBB/BTB modulation using vascular-targeted gold nanoparticles and pulsed laser excitation has shown preclinical potential to enhance paclitaxel delivery, reduce tumor burden, and prolong survival in GBM models that recapitulate angiogenic-core and infiltrative-margin phenotypes; however, this approach remains preclinical and is best positioned as a complementary barrier-modulation strategy rather than albumin-specific evidence [43]. Future studies need to optimize the synchronization of albumin-based therapeutic administration with BBB/BTB-opening parameters, including treatment timing, acoustic or optical dose, treatment volume, imaging-confirmed barrier opening, spatial pharmacokinetic sampling, repeated-procedure safety, payload-specific toxicity, and biomarker-guided patient selection. BBB modulation should therefore be developed as a controlled delivery-enhancement strategy rather than a nonspecific permeability enhancer [69].

### 9.2. Biomarker-Driven Patient Selection

Albumin-based therapeutics are unlikely to benefit all patients uniformly, making biomarker-guided patient selection and payload-specific stratification essential. For albumin-bound paclitaxel, SSR3 currently has the strongest payload-specific evidence among proposed sensitivity markers, as CRISPR-based screening and analyses in an orthotopic glioma model identified the translocon-associated protein subunit SSR3 as a candidate predictor of paclitaxel susceptibility [60]. Albumin-receptor profiling is better positioned as an exploratory enrichment and mechanism-validation strategy than as an established patient-selection tool, because gp60/albondin, SPARC, FcRn, and caveolin-associated pathways may influence albumin transport, recycling, and tumor retention but have not been validated as universal predictors of clinical response [10,12]. P-glycoprotein and related efflux transporters are best considered delivery-resistance variables rather than simple tumor-cell sensitivity biomarkers, particularly for payloads susceptible to efflux-mediated restriction, given their central role in limiting CNS drug exposure across the BBB [5]. IRE1α may serve as an exploratory stress-adaptation and resistance-associated biomarker, although current evidence in glioblastoma is stronger for temozolomide sensitization than for direct prediction of albumin-bound paclitaxel response [61]. Dynamic liquid biopsy may further support response monitoring, particularly as focused ultrasound-based BBB opening becomes clinically feasible and sonobiopsy strategies increase the release or detectability of tumor-derived circulating biomarkers in peripheral blood [67,70].

### 9.3. Synergistic Combination Therapies

Combination strategies should be prioritized based on defined resistance biology, payload-specific pharmacology, and microenvironmental context rather than on empirical drug pairing. For albumin-bound paclitaxel, the most direct preclinical rationale remains temozolomide sensitization through disruption of DNA-damage repair and ferroptosis-associated vulnerability [45]. This interaction supports a payload-specific combination hypothesis, but it should not be generalized to all albumin formulations or all GBM resistance states.

Future albumin-based combinations should meet three translational requirements. First, the payload combination should address a defined GBM vulnerability, such as DNA-repair adaptation, efflux-associated underexposure, immune exclusion, or metabolic stress adaptation. Second, albumin-based delivery should solve a specific pharmacologic limitation, such as poor solubility, insufficient regional exposure, inadequate tumor retention, or the need to synchronize systemic therapy with BBB/BTB opening. Third, the combination should demonstrate payload-specific target engagement and acceptable neurologic safety in models that preserve BBB/BTB heterogeneity and tumor–immune context.

Accordingly, combinations with temozolomide, radiotherapy, immunotherapy, ferroptosis-inducing approaches, metabolic interventions, or pathway-directed agents should be advanced only when spatial pharmacokinetics, pharmacodynamic biomarkers, and toxicity profiles support a coherent mechanism–exposure–efficacy relationship. Patient-derived organoids, tumor–immune co-cultures, and orthotopic models may help prioritize these combinations before clinical testing, as illustrated by real-time patient-derived GBM organoid platforms used to assess CAR-T cell bioactivity in parallel with clinical treatment; however, clinical development should ultimately require biomarker-informed selection and patient-centered endpoints, including survival, neurologic safety, and quality of life [10,62,66].

### 9.4. Advanced Formulation Development

The next generation of albumin-based GBM carriers needs to solve a problem that current formulations have not addressed adequately: converting systemic drug delivery into spatially controlled intratumoral release. Stimuli-responsive designs—nanoparticles that remain intact in circulation but disassemble in response to tumor-associated cues such as acidic pH, elevated glutathione, or protease activity—offer one route toward this goal. However, demonstrating selective stimulus-responsive release at relevant tumor sites, rather than premature release in blood or non-target tissue, remains a substantial validation challenge [10]. Layering active targeting ligands onto the albumin surface—for instance, angiopep-2 peptides to exploit LRP1-mediated transcytosis—introduces additional specificity but also additional failure modes: receptor saturation, interpatient expression variability, and off-target uptake in brain endothelium all need systematic prospective evaluation before ligand-functionalized albumin carriers can be considered ready for clinical development [5]. Incorporating imaging reporters into the same construct would at least allow non-invasive tracking of these failure modes, which is why theranostic albumin platforms, though still largely preclinical, warrant further systematic evaluation [13]. Formulation development should define critical quality attributes, including particle size, polydispersity, drug loading, albumin conformation, release kinetics, receptor-binding integrity, sterility, endotoxin burden, and storage stability [10,13].

### 9.5. Manufacturing Innovation

Clinical translation depends on manufacturing strategies that can produce albumin-based nanomedicines with batch-to-batch consistency, scalable throughput, and regulatory-grade quality control. The broader nanomedicine field has identified inadequate physicochemical characterization, limited reproducibility, scale-up constraints, good manufacturing practice (GMP) implementation, and regulatory uncertainty as recurring barriers to clinical translation [13]. Continuous-flow or microfluidic manufacturing may improve control over mixing, nanoparticle formation, size distribution, and process reproducibility, making these approaches attractive for scalable albumin nanoparticle production [13]. Green or solvent-minimized synthesis approaches are preferable when they preserve albumin structure, avoid toxic residual solvents, and maintain drug-loading efficiency [10]. For clinical-grade development, manufacturing optimization needs early integration with analytical release testing, stability protocols, sterility assurance, residual-solvent quantification, and comparability assessment after scale-up [10,13].

## 10. Positioning Albumin-Based Drug Delivery Among Competing and Complementary GBM Delivery Strategies

### 10.1. Comparative Advantages and Limitations of Albumin-Based Nanoparticles Relative to Other Nanocarrier Platforms

Albumin-based nanoparticles occupy a distinctive position among glioblastoma drug delivery platforms. Their main advantages are endogenous carrier biology, hydrophobic drug-binding capacity, biologically plausible albumin-associated transport or retention mechanisms, and substantial clinical formulation precedent. Albumin can interact with gp60/albondin, SPARC, FcRn, and caveolin-associated pathways, potentially supporting endothelial transcytosis, tumor retention, recycling, and vesicular transport under specific biological conditions. These pathways are best regarded as context-dependent mechanistic contributors rather than validated universal predictors of clinical response [10,12]. Albumin-associated biology does not eliminate the BBB/BTB barrier, but it may enhance tumor exposure when receptor expression, vascular accessibility, and intratumoral retention are favorable [5].

Polymeric nanoparticles, lipid nanoparticles, and viral vectors each offer complementary advantages, and their value depends on the therapeutic objective rather than a single ranking criterion. Polymeric nanoparticles provide broad chemical tunability, controlled-release potential, and payload flexibility, although translation may be limited by formulation complexity, process sensitivity, and scale-up reproducibility [13,27]. Lipid nanoparticles have transformed nucleic acid delivery, particularly for messenger RNA (mRNA) and small interfering RNA (siRNA) therapeutics, but efficient systemic RNA delivery does not, by itself, ensure adequate brain tumor accumulation without BBB-targeting or BBB-opening strategies [5,71]. Viral vectors offer high transduction efficiency and are uniquely suited for gene delivery, although their use is constrained by immunogenicity, payload limits, manufacturing complexity, tropism, redosing limitations, and long-term safety considerations [72].

Table 5 presents a condensed comparison of albumin-based nanoparticles with major alternative nanocarrier and vector platforms. A more detailed characteristic-by-platform comparison is provided in Supplementary Table S2.

This comparison is intended to clarify platform fit rather than to rank albumin-based nanoparticles as universally superior. Their principal strengths lie in clinical formulation maturity, endogenous carrier biology, compatibility with hydrophobic or albumin-binding payloads, and biologically plausible linkage to albumin-associated transport or retention pathways that require GBM-specific validation [10,12]. By comparison, polymeric nanoparticles may provide greater structural and release-profile flexibility, lipid nanoparticles remain especially powerful for RNA delivery, and viral vectors are uniquely suited for gene-transfer applications [27,71,72]. In glioblastoma, albumin-based systems are most compelling when the therapeutic goal is to improve brain-tumor exposure of albumin-compatible therapeutic or imaging payloads within a clinically translatable formulation framework. Their use should still avoid the assumption that albumin-associated transport alone is sufficient for clinical efficacy [10,14].

### 10.2. Complementarity Between Albumin-Based Delivery and Physical BBB/BTB-Modulation or Bypass Strategies

Physical strategies for overcoming BBB/BTB-limited delivery, including low-intensity pulsed ultrasound with microbubbles, MR-guided focused ultrasound, optical BBB/BTB modulation, and convection-enhanced delivery, directly address inadequate drug access to the tumor-infiltrated brain, a central barrier in glioblastoma therapy [14,69]. These methods can increase regional drug entry, but their performance depends on spatial coverage, treatment planning, imaging confirmation, procedural repeatability, infrastructure requirements, and safety monitoring [69]. Convection-enhanced delivery bypasses systemic BBB limitations through direct intraparenchymal infusion. However, clinical implementation remains technically demanding and depends heavily on catheter placement, tissue anisotropy, infusion direction, distribution volume, leakage control, and tumor coverage [73,74]. These approaches differ substantially in their evidence level and translational role. Recent clinical studies using implantable ultrasound with carboplatin and transcranial microbubble-enhanced focused ultrasound with temozolomide further support the feasibility of repeated or localized BBB opening in glioma, although these studies provide platform-level evidence rather than albumin-specific validation [67,75]. LIPU/MB combined with albumin-bound paclitaxel currently provides the most direct albumin-specific human CNS pharmacokinetic evidence, whereas MR-guided or transcranial focused ultrasound, optical BBB/BTB modulation, and convection-enhanced delivery (CED) provide broader platform-level or supportive evidence for improving regional access to GBM tissue rather than direct validation of albumin-based delivery [14].

Albumin-based delivery and physical BBB/BTB modulation are therefore better viewed as complementary rather than competing strategies. BBB-opening technologies can transiently increase regional access to the brain, whereas albumin-based carriers may improve drug solubilization, systemic persistence, payload exposure, and intratumoral retention after barrier modulation. Receptor-associated uptake remains a context-dependent mechanism that requires validation rather than being an assumed determinant of efficacy [12,14]. This distinction is clinically important because BBB opening alone cannot ensure adequate tumor-cell exposure when the administered drug has poor solubility, rapid systemic clearance, limited tissue retention, unfavorable pharmacokinetics, or insufficient cellular susceptibility [5].

The ANG1005 experience reinforces this principle from a non-albumin perspective: LRP1-mediated BBB transcytosis and an acceptable taxane-class safety profile were insufficient to achieve prespecified efficacy in recurrent high-grade glioma [55]. For albumin-based delivery, the same lesson applies: BBB transport enhancement must be integrated with tumor retention, spatial penetration, payload susceptibility, and biomarker-guided patient selection. Conversely, albumin-based delivery alone may be insufficient in tumor regions with relatively intact BBB, spatially heterogeneous albumin-receptor expression, or inadequate payload retention and tumor-cell susceptibility [6,12].

The strongest translational rationale is an integrated approach that combines controlled BBB/BTB opening with albumin-compatible therapeutics, pairs delivery enhancement with spatial pharmacokinetic validation, and assesses pharmacodynamic and patient-centered outcomes. As summarized in Section 6, LIPU/MB-mediated BBB opening with albumin-bound paclitaxel provides the most direct GBM-specific proof of concept for pairing a physical delivery method with an albumin-based formulation, because it links controlled BBB opening to measurable human CNS tissue pharmacokinetics. However, this evidence should be interpreted as feasibility, safety, and pharmacokinetic enhancement rather than definitive therapeutic validation [14]. Optical BBB/BTB modulation using vascular-targeted gold nanoparticles and pulsed laser excitation has also shown preclinical potential to enhance paclitaxel delivery in GBM models that capture vascular and infiltrative tumor compartments; however, this evidence supports complementary barrier modulation rather than albumin-specific delivery [43]. These findings favor a development model in which albumin-carrier design, payload selection, barrier-opening parameters, imaging-confirmed delivery, spatial pharmacokinetic sampling, safety monitoring, and exploratory patient-selection biomarkers are optimized together rather than independently [13,69]. Accordingly, the choice of BBB/BTB-modulation strategy should be guided by the target tumor compartment, required spatial coverage, timing between barrier opening and systemic drug exposure, compatibility with repeat dosing, and the safety profile of both the device and payload.

In summary, the translational value of combining albumin-based delivery with physical BBB/BTB modulation lies in aligning systemic formulation advantages with controlled regional brain access. This approach may be particularly relevant in glioblastoma, where therapeutic failure reflects not only inadequate barrier penetration but also insufficient payload retention, spatially heterogeneous tumor compartments, and variable tumor-cell susceptibility. However, its clinical adoption should depend on prospective evidence that improved delivery translates into meaningful clinical benefit while preserving neurologic safety and enabling biomarker- or imaging-guided patient selection [6,14].

## 11. Conclusions and Summary

Albumin-based drug delivery provides a clinically mature and mechanistically plausible approach for improving the pharmacological behavior of selected therapeutics in glioblastoma, with nanoparticle albumin-bound paclitaxel as the most extensively developed example. The rationale is grounded in albumin’s endogenous carrier biology, hydrophobic drug-binding capacity, FcRn-mediated persistence, and potential engagement of gp60/albondin-, SPARC-, FcRn-, and caveolin-associated pathways [9,11,12]. These mechanisms provide a rational delivery framework, although their current role is best defined as context-dependent contribution rather than validated universal prediction of GBM response [12,24].

Preclinical studies indicate that albumin-based formulations can improve brain-tumor drug exposure, enhance intratumoral accumulation, and support rational combination strategies. Engineered albumin nanoparticles have been used to target SPARC- and gp60-associated pathways to improve BBB penetration and glioma uptake, and paclitaxel/fenretinide co-loaded albumin nanoparticles showed antitumor activity in intracranial glioma models [21]. Albumin-bound paclitaxel has also enhanced temozolomide sensitivity by intensifying DNA-damage signaling, impairing repair programs, and promoting ferroptosis-associated vulnerability in GBM models [45].

The most direct GBM-specific clinical evidence is still early, but it is clinically informative. Repeated LIPU/MB-mediated BBB opening using an implantable ultrasound device enhanced peritumoral delivery of albumin-bound paclitaxel in recurrent glioblastoma. It provided human proof of concept for combining albumin-bound chemotherapy with regional BBB modulation [14]. This evidence primarily supports feasibility, safety, and pharmacokinetic enhancement, not definitive survival benefit. Likewise, the clinical success of nab-paclitaxel in metastatic breast cancer, advanced non-small cell lung cancer, and metastatic pancreatic adenocarcinoma establishes formulation maturity and systemic dosing experience. However, it does not, by itself, establish efficacy in GBM [33,49,51].

Several barriers remain decisive for clinical translation. Spatially heterogeneous BBB/BTB permeability, invasive-margin protection, adaptive resistance, and immunosuppressive tumor biology can reduce the therapeutic impact of even well-designed delivery systems [5,6,7]. Future development requires an integrated strategy that combines BBB/BTB-opening technologies, spatial pharmacokinetic sampling, receptor-pathway validation, patient-derived models, biomarker-guided enrichment, and payload-specific combinations selected according to mechanism–exposure–efficacy relationships.

At present, albumin-based delivery is best positioned as a promising but clinically unproven strategy in the GBM therapeutic landscape. The mechanistic rationale is coherent, the formulation precedent from extracranial cancers is mature, and the LIPU/MB phase I trial has produced the first human evidence that albumin-bound paclitaxel can achieve measurable peritumoral brain exposure when the BBB is transiently opened. What remains absent is the evidence that matters most clinically: a randomized trial showing that this pharmacokinetic enhancement translates into improved survival, preserved neurocognitive function, or meaningful quality-of-life benefit. Until such evidence is available, the most defensible role of albumin-based delivery is as one pharmacologically rational component within integrated, biomarker-stratified strategies that address the biological, spatial, and pharmacological complexity of GBM.

The next phase of albumin-based GBM therapeutics should therefore be judged not by tumor accumulation alone, but by spatially resolved pharmacokinetic validation, invasive-margin exposure, payload-specific susceptibility, neurologic tolerability, and clinically meaningful patient outcomes.

## Figures and Tables

**Table 1:** Principal albumin-associated uptake, transport, recycling, and retention mechanisms relevant to glioblastoma drug delivery.

Mechanism	Molecular Axis	Principal Biological Role	Relevance to GBM Delivery	Normal-Tissue or Off-Target Implication
Caveola-mediated endothelial transcytosis	gp60/albondin–Src-family kinase–caveolin-1	Vesicular endothelial albumin transport [19,20]	May support albumin-associated endothelial crossing in selected BBB/BTB contexts [12,21]	Not GBM-selective; may also operate in normal or inflamed vascular beds [12,19]
Caveolin-associated vesicular trafficking	Caveolin-1, caveolae, endothelial vesicles	Transcellular vesicle formation and trafficking [26]	May contribute to endothelial transport but does not bypass tight-junction restriction in BBB-preserved margins [5]	Broad endothelial process; not tumor-specific [26]
Tumor- and stroma-associated retention	SPARC–albumin interaction	Albumin binding, matrix remodeling, stromal retention [12,22]	May enhance retention in selected SPARC-rich glioma or stromal niches [21,23]	Context-dependent; not a validated universal biomarker [24]
Systemic recycling and persistence	FcRn–albumin interaction	Endosomal rescue from lysosomal degradation and systemic recycling [11,17]	May prolong exposure before tumor vascular encounter [16]	Extends systemic exposure but does not ensure tumor selectivity [10]
Ligand-directed BBB or tumor targeting	LRP1/Angiopep-2, integrins/cRGD, other ligands	Engineered receptor-mediated uptake or transcytosis [5,27]	Relevant mainly to modified albumin constructs [27]	Limited by receptor heterogeneity, saturation, and off-target uptake [5]
Immune-cell uptake and cell-mediated transport	Macrophage/myeloid uptake, macropinocytosis, cell-loaded albumin-bound drug	Myeloid uptake, immune interaction, or cell-mediated carriage [28,29]	May support macrophage-assisted delivery in experimental GBM systems [29]	GBM microglia may differ from peripheral macrophages [30]
Passive extravasation through disrupted BTB	Leaky tumor vasculature, EPR-like accumulation	Nonspecific macromolecular or nanoparticle extravasation [25]	May contribute to enhancing core accumulation [7]	Does not reflect albumin-specific transport and may spare BBB-preserved invasive margins [6]

**Table 2:** Representative clinical precedents and brain tumor-relevant clinical studies of albumin-based therapeutics.

Category	Trial/Therapeutic Context	Relevance	Evidence Level/Role	References
Extracranial taxane platform precedent	NCT00046527; metastatic breast cancer; nab-paclitaxel vs. solvent-based paclitaxel; phase III	Established solvent-free albumin-bound paclitaxel as a clinically feasible taxane formulation.	Clinical efficacy evidence in extracranial cancer; formulation/safety precedent for GBM translation	[33,47]
Extracranial taxane combination precedent	NCT00540514; advanced NSCLC; nab-paclitaxel plus carboplatin; phase III	Supported use of nab-paclitaxel in systemic combination chemotherapy.	Clinical efficacy evidence in extracranial cancer; systemic combination precedent	[48,49]
Extracranial combination and biomarker precedent	NCT00844649; metastatic pancreatic adenocarcinoma; nab-paclitaxel plus gemcitabine; phase III	Demonstrated combination efficacy and cautioned against SPARC as a universal predictive biomarker.	Clinical efficacy evidence in extracranial cancer; biomarker caution for SPARC	[24,50,51]
GBM-specific albumin-bound taxane delivery	NCT04528680; recurrent glioblastoma; LIPU/MB-mediated BBB opening plus albumin-bound paclitaxel; phase I/II	Provided human proof-of-concept for enhanced peritumoral CNS delivery.	Clinical pharmacokinetic evidence in GBM; no definitive efficacy evidence	[14,52]
Brain tumor albumin-bound mTOR inhibitor strategy	NCT03463265; recurrent high-grade glioma and newly diagnosed GBM; nab-sirolimus/ABI-009/nab-rapamycin; phase II	Extended albumin-bound therapeutic development beyond taxane delivery.	Exploratory clinical brain tumor evidence; efficacy unproven	[53]
Pediatric CNS-relevant albumin-bound mTOR inhibitor precedent	NCT02975882; pediatric recurrent or refractory solid tumors, including CNS tumors; nab-sirolimus plus temozolomide and irinotecan; phase I	Established dose-limiting toxicity, pharmacokinetic, and recommended dosing framework for nab-sirolimus combination therapy.	CNS-relevant clinical safety/pharmacokinetic precedent; non-GBM-specific efficacy	[54]

**Table 3:** Concise translational go/no-go decision framework for albumin-based drug delivery in glioblastoma.

Decision Domain	Go Signal	No-Go or Caution Signal	Key Evidence to Prioritize	Representative References
Formulation readiness	Reproducible albumin formulation with defined payload loading, stability, release behavior, and manufacturability	Aggregation, unstable payload binding, variable batch performance, or poorly defined release kinetics	CMC reproducibility, stability testing, release profiling, impurity control	[8,10,13]
Spatial delivery and therapeutic window	Increased tumor or peritumoral exposure with acceptable normal-brain and systemic safety	Exposure limited to permeable tumor core, inadequate invasive-margin delivery, or disproportionate normal-brain toxicity	Spatial pharmacokinetics, tumor-to-normal brain exposure, invasive-margin sampling, neurologic safety	[6,7,14,34]
Mechanistic and payload validation	Functional evidence that albumin-associated transport, retention, or payload activity contributes to efficacy in the relevant GBM context	Receptor expression or uptake is assumed without pathway validation, or delivery occurs without payload target engagement	Receptor/pathway profiling, blocking or knockdown studies, pharmacodynamic biomarkers, patient-derived models	[12,23,24,45]
Clinical benefit threshold	Delivery enhancement is linked to radiographic, survival, neurocognitive, quality-of-life, or biomarker-enriched benefit	Delivery success does not translate into durable response, survival benefit, or acceptable patient-centered outcomes	Response Assessment in Neuro-Oncology (RANO)-based response, progression-free survival (PFS)/overall survival (OS), neurocognitive outcomes, quality-of-life measures, biomarker-guided subgroup analysis	[10,14,55]

**Table 4:** A condensed framework of advanced preclinical models for evaluating albumin-based drug delivery systems in glioblastoma.

Model Platform	Best-Suited Translational Question	Key Evaluation Endpoints for Albumin-Based GBM Delivery
Patient-derived orthotopic xenografts, patient-derived orthotopic xenograft (PDOX)/patient-derived xenograft (PDX)	Does the albumin-based formulation improve brain-tumor exposure and survival in an orthotopic patient-derived context?	Tumor-to-normal brain accumulation, brain/tumor pharmacokinetics, invasive-margin penetration, survival benefit, inter-patient response variability, and preservation of molecular heterogeneity [14,65]
Patient-derived GBM organoids and ex vivo tumor slices	Does the carrier penetrate patient-specific tumor tissue and preserve individualized drug-response patterns?	Organoid or tissue penetration kinetics, surface-to-core delivery gradient, hypoxia-associated drug response, individualized sensitivity, and preservation of tumor cytoarchitecture [64,65]
Tumor–immune organoid co-cultures and humanized orthotopic models	Does albumin-based delivery interact with human immune compartments or support immunotherapy combinations?	Albumin-carrier uptake by macrophage/myeloid compartments, tumor–immune spatial interactions, cytokine response, T-cell dynamics, CAR-T or checkpoint-combination response, and immunotoxicity [62,66]
Immunocompetent syngeneic and genetically engineered mouse models (GEMMs)	How do intact immune and stromal compartments influence biodistribution, efficacy, and toxicity?	Brain accumulation under intact immunity, tumor-associated macrophage (TAM)/microglia infiltration, CD8/regulatory T cell (Treg)/myeloid-derived suppressor cell (MDSC) balance, BBB/BTB remodeling during tumor evolution, invasion-associated delivery, and native stromal co-evolution [39,62]
BBB/BTB–GBM-on-a-chip and vascularized 3D GBM models	Can the carrier cross a controlled BBB/BTB interface and accumulate on the tumor side?	Transendothelial electrical resistance (TEER) or barrier-integrity readouts, permeability assays, transcytosis rate, tumor-side nanocarrier accumulation, vascular leakage, extravasation, ECM stiffness-dependent diffusion, and real-time delivery kinetics [64]
Post-treatment and recurrence-mimicking models	Does therapy-induced vascular or immune remodeling alter albumin-carrier delivery at recurrence?	Delivery to BBB-intact invasive margins, recurrence-site accumulation, therapy-induced changes in SPARC/gp60/FcRn/caveolin expression, recurrent TME phenotype, and recurrence-free survival [2,39]
Receptor-validated human tissue–model matching	Does the model reproduce human GBM albumin-transport biology?	SPARC, gp60/albondin, FcRn, and caveolin-1 profiling; receptor-positive niche mapping; immunohistochemistry (IHC) or spatial transcriptomics-based vessel/tumor colocalization; and model-to-patient concordance scoring [10,12]
Device-assisted BBB/BTB modulation models	Can albumin-based delivery be synchronized with clinically relevant BBB-opening parameters?	Focused ultrasound- or implantable ultrasound-mediated BBB opening, peritumoral delivery enhancement of albumin-bound paclitaxel, BBB-opening safety, regional delivery mapping, and synchronization of drug administration with barrier modulation [14]

**Table 5:** Condensed comparative positioning of albumin-based nanoparticles relative to alternative nanocarrier platforms for glioblastoma drug delivery.

Platform	Principal Strength	Major Limitation in GBM	Most Suitable Application Context
Albumin-based nanoparticles	Endogenous protein carrier with hydrophobic drug-binding capacity, established formulation precedent, and biologically plausible albumin-associated transport or retention pathways requiring context-specific validation [10,12]	Delivery remains dependent on BBB/BTB status, albumin-receptor expression, vascular accessibility, and spatial tumor heterogeneity [5]	Hydrophobic anticancer drugs, albumin-binding payloads, imaging agents, and BBB-opening combinations when albumin-associated transport or retention can be mechanistically validated [10,12,14]
Polymeric nanoparticles	Broad chemical tunability, controlled-release capability, and flexible payload engineering [27]	Translation can be limited by formulation complexity, heterogeneous tumor penetration, process sensitivity, and scale-up reproducibility [13]	Programmable release systems, ligand-functionalized carriers, combination payloads, and precision nanoparticle engineering, where physicochemical properties and manufacturing reproducibility can be controlled [13,27]
Lipid nanoparticles	Strong clinical precedent for RNA delivery, particularly mRNA and siRNA therapeutics [71]	Passive CNS entry is limited; brain-tumor accumulation usually requires active targeting, local delivery, or BBB-opening approaches [5]	mRNA, siRNA, gene-editing cargos, and immune-modulatory nucleic-acid therapeutics when combined with targeting strategies or BBB/BTB-modulation approaches for CNS delivery [5,71]
Viral vectors	High gene-transfer efficiency and suitability for gene-replacement or gene-modifying strategies [72]	Immunogenicity, payload constraints, manufacturing complexity, tropism, redosing limitations, and long-term safety concerns [72]	Gene therapy, gene editing, cellular reprogramming, and selected CNS-targeted genetic interventions requiring durable transgene expression or cell-type-specific transduction [5,72]

## Data Availability

No new data were created or analyzed in this study.

## Supplementary Materials

The following supporting information can be downloaded at: https://www.mdpi.com/article/10.3390/cells15131180/s1, Table S1: Expanded preclinical model-by-endpoint framework for evaluating albumin-based drug delivery systems in glioblastoma [6,10,12,14,39,62,64,65,66]; Table S2: Detailed comparison of albumin-based nanoparticles, polymeric nanoparticles, lipid nanoparticles, and viral vectors for glioblastoma drug delivery [5,10,12,13,14,27,71,72].

## Abbreviations

The following abbreviations are used in this manuscript:

BBB	Blood–brain barrier
BTB	Blood–tumor barrier
CAR-T	Chimeric antigen receptor T-cell
CBTRUS	Central Brain Tumor Registry of the United States
CED	Convection-enhanced delivery
cGAS	Cyclic GMP–AMP synthase
CMC	Chemistry, manufacturing, and controls
CNS	Central nervous system
CRISPR	Clustered regularly interspaced short palindromic repeats
cRGD	Cyclic arginine–glycine–aspartic acid
ECM	Extracellular matrix
EORTC	European Organisation for Research and Treatment of Cancer
EPR	Enhanced permeability and retention
FcRn	Neonatal Fc receptor
GBM	Glioblastoma
GEMM	Genetically engineered mouse model
GMP	Good manufacturing practice
gp60	Glycoprotein 60
HSA	Human serum albumin
HMGB1	High-mobility group box 1
IDH	Isocitrate dehydrogenase
IHC	Immunohistochemistry
IL	Interleukin
IRE	Inositol-requiring enzyme
KU70	Ku70 DNA repair protein
LIPU/MB	Low-intensity pulsed ultrasound with microbubbles
LMWP	Low-molecular-weight protamine
LRP1	Low-density lipoprotein receptor-related protein 1
MDSC	Myeloid-derived suppressor cell
MPACT	Metastatic Pancreatic Adenocarcinoma Clinical Trial
MR	Magnetic resonance
mTOR	Mechanistic target of rapamycin
nab	Nanoparticle albumin-bound
NCIC	National Cancer Institute of Canada
NSCLC	Non-small cell lung cancer
optoBBTB	Optical blood–brain–tumor barrier
OS	Overall survival
PDOX	Patient-derived orthotopic xenograft
PDX	Patient-derived xenograft
PEG	Polyethylene glycol
PFS	Progression-free survival
PK	Pharmacokinetic
PRISMA	Preferred Reporting Items for Systematic Reviews and Meta-Analyses
RANO	Response Assessment in Neuro-Oncology
RGD	Arginine–glycine–aspartic acid
RNA	Ribonucleic acid
ROS	Reactive oxygen species
siRNA	Small interfering RNA
SPARC	Secreted protein acidic and rich in cysteine
SSR3	Signal sequence receptor subunit 3
STAT3	Signal transducer and activator of transcription 3
STING	Stimulator of interferon genes
TAM	Tumor-associated macrophage
TEER	Transendothelial electrical resistance
TLR4	Toll-like receptor 4
TME	Tumor microenvironment
TRAIL	TNF-related apoptosis-inducing ligand
Treg	Regulatory T cell
