# Supplementary material for: Albumin-Based Drug Delivery for Glioblastoma Treatment: Mechanistic Rationale, Preclinical Evidence, and Clinical Translation
Source: Cells. 2026 Jun 29;15(13):1180. doi: 10.3390/cells15131180 (PMC13359503; doi:10.3390/cells15131180)
Supplement: Supplementary file 1 [file cells-15-01180-s001.zip › cells-4379705-supplementary.pdf]

**Supplementary Table S1. Expanded preclinical model-by-endpoint framework for evaluating albumin-based drug delivery systems in glioblastoma**

This supplementary table provides an expanded endpoint matrix for advanced preclinical platforms used to evaluate albumin-based GBM drug delivery systems. It includes patient-derived orthotopic xenografts, GBM organoids, tumor–immune co-cultures, syngeneic and humanized orthotopic models, GEMMs, BBB/BTB–GBM-on-a-chip systems, vascularized 3D GBM models, ex vivo brain/tumor slice cultures, post-treatment or recurrence-mimicking models, receptor-validated human tissue–model matching, and device-assisted BBB/BTB modulation models. For each platform, the table details BBB/BTB and PK/biodistribution endpoints, albumin-specific mechanism endpoints, spatial penetration and invasion endpoints, therapeutic efficacy readouts, TME/immune-fidelity indicators, and safety or translational-readiness considerations. It is intended to support rational model selection and endpoint prioritization rather than to rank experimental systems.

| Model platform | Primary purpose | Key BBB/BTB and biodistribution endpoints | Albumin-specific mechanism endpoints | Spatial and invasive-margin endpoints | Therapeutic and translational endpoints |
|---|---|---|---|---|---|
| Patient-derived orthotopic xenograft, PDOX/PDX | Reproduce patient-derived GBM heterogeneity and orthotopic brain growth | Tumor-to-normal brain accumulation ratio; brain/tumor pharmacokinetic exposure [14] | SPARC/gp60/FcRn/caveolin-1 expression profiling; receptor-blocking or knockdown validation [10,12] | Invasive-margin delivery; tumor penetration depth [6] | Tumor-burden reduction, survival extension, patient-to-patient response variability, and molecular heterogeneity preservation [65] |
| Patient-derived GBM organoids/explant organoids | Model patient-specific drug response and preserve tumor architecture ex vivo | Organoid penetration kinetics; patient-specific drug-response screening [65] | SPARC/gp60/FcRn/caveolin-1 profiling before treatment; albumin-carrier uptake quantification [12] | Surface-to-core penetration gradient; hypoxia-associated delivery gradient [65] | Viability reduction, preservation of tumor architecture and cellular diversity, and feasibility for precision drug screening [65] |
| Autologous tumor–immune organoid co-culture | Evaluate patient-matched human tumor–immune interactions | Immune-cell-dependent modulation of nanocarrier penetration; immune-compartment uptake [66] | Albumin-carrier uptake by macrophage or myeloid compartments; SPARC/gp60-associated uptake under immune co-culture [12] | Tumor–immune spatial interaction mapping; organoid invasion into brain-like tissue contexts [66] | Immune-mediated tumor killing, CAR-T or immune-combination response, T-cell clonotype dynamics, and cytokine-response monitoring [66] |
| Immunocompetent syngeneic orthotopic GBM models | Test albumin-based delivery in an intact immune system | Brain accumulation under intact murine immunity; systemic immune uptake and clearance [64] | Species-specific albumin-receptor compatibility; murine SPARC/gp60 comparison [12] | Tumor-margin accumulation; infiltrative growth assessment [6] | Survival extension, tumor-volume reduction, TAM/microglia infiltration, CD8/Treg/MDSC balance, and model-dependent immunotherapy response [62] |

| | | | | | |
|---|---|---|---|---|---|
| Humanized orthotopic GBM models | Evaluate human immune responses to albumin-based carriers | Human immune-cell impact on biodistribution; tumor infiltration of human immune cells [64] | Human albumin-receptor compatibility; human SPARC/gp60/FcRn validation [12] | Human immune-cell distribution within tumor regions; tumor–immune spatial architecture [39] | Checkpoint blockade or CAR-T combination response, human myeloid-dominant immune landscape, and incomplete human microglia/BBB reconstruction as a limitation [62] |
| Genetically engineered mouse models, GEMMs | Model de novo GBM evolution with native brain stroma and immune system | BBB/BTB remodeling during tumor evolution; longitudinal delivery during tumor progression [6] | Albumin-receptor expression over tumor evolution; caveolin/gp60-associated transcytosis assessment [12] | Invasion-associated delivery pattern; angiogenesis-associated accumulation [6] | Survival benefit, tumor-progression delay, native immune/stromal co-evolution, and mechanistic value despite limited patient-specific heterogeneity [39] |
| BBB/BTB–GBM-on-a-chip | Quantify BBB/BTB crossing and tumor-side delivery under controlled microfluidic conditions | TEER or barrier-integrity readout; FITC-dextran permeability assay; transcytosis rate; device-assisted BBB-modulation readout [64] | gp60-mediated endothelial transport; SPARC-mediated tumor retention [12] | Tumor-side nanocarrier accumulation; real-time delivery kinetics after ultrasound or sonodynamic stimulation [64] | Tumor-cell killing in a barrier-protected setting, treatment-response kinetics, high-throughput screening potential, and incomplete whole-brain immune/ECM complexity as a limitation [64] |
| Vascularized 3D bioprinted GBM models | Reproduce vascularized, ECM-rich, spatially heterogeneous GBM tissue | Vascular leakage, extravasation, and perfusable vessel-associated delivery [64] | Endothelial albumin-transport marker analysis; albumin-carrier uptake in vascularized regions [10] | ECM stiffness-dependent diffusion, oxygen-gradient-associated penetration, and spatially resolved receptor-positive niches [39] | Drug response in patient-specific 3D constructs, tumor-killing efficacy under vascularized conditions, and fabrication reproducibility/scale-up limitations [64] |
| Organotypic brain slice/ex vivo GBM slice culture | Preserve native brain architecture, ECM, and invasive-edge biology | Local diffusion and retention in native tissue; ex vivo delivery across preserved brain matrix [64] | SPARC/gp60 expression in native tissue context; albumin-carrier uptake in patient tissue context [12] | Tumor-cell migration distance; invasive-margin accumulation; spatial transcriptomics-based receptor-niche mapping [6,39] | Short-term therapeutic response, individualized treatment response, native cytoarchitecture preservation, and limited culture viability as a limitation [65] |
| Post-treatment / recurrence-mimicking models | Evaluate delivery to residual and recurrent GBM after surgery, radiotherapy, or temozolomide. | Drug delivery to BBB-intact invasive margins; delivery after radiation- or ultrasound-induced | Treatment-induced changes in SPARC/gp60/FcRn expression; albumin-bound drug uptake after | Residual-cell targeting at invasive margins; recurrence-site accumulation [6] | Recurrence-free survival, therapy-driven ecosystem evolution, recurrent TME phenotype, and standardized recurrence definitions [39] |

| | | | | | |
|---|---|---|---|---|---|
| | | BBB/BTB modulation [6,14] | standard-of-care exposure [12] | | |
| Receptor-validated human tissue–model matching | Confirm whether the selected model reflects albumin-transport biology in human GBM | Matched tissue/model biodistribution concordance; tumor-to-normal brain delivery benchmark [14] | SPARC, gp60/albondin, FcRn, and caveolin-1 expression profiling [10,12] | Spatial transcriptomics-based receptor-positive niche mapping; IHC-based vessel/tumor colocalization [39] | Go/no-go translational decision-making, response stratification by albumin-receptor status, and avoidance of overinterpretation from non-representative models [10] |
| Device-assisted BBB/BTB modulation models | Evaluate albumin-based carriers under clinically relevant BBB-opening strategies, especially focused ultrasound or implantable ultrasound devices. | Focused ultrasound-mediated BBB opening; albumin-bound paclitaxel delivery to peritumoral brain; BBB-opening safety and reversibility [14] | Albumin-bound drug compatibility with BBB-opening platforms; receptor-mediated uptake after barrier modulation [12] | Peritumoral delivery enhancement; region-specific BBB-opening map [14] | Enhanced chemotherapy exposure, standardized ultrasound dose/timing/imaging endpoints, and direct clinical translational relevance [14] |

**Supplementary Table S2. Detailed comparison of albumin-based nanoparticles, polymeric nanoparticles, lipid nanoparticles, and viral vectors for glioblastoma drug delivery**

This supplementary table provides a detailed characteristic-by-platform comparison of albumin-based nanoparticles, polymeric nanoparticles, lipid nanoparticles, and viral vectors. The table expands the condensed comparison presented in Table 5 by evaluating core advantages, biocompatibility, BBB/BTB delivery potential, payload suitability, clinical precedent, manufacturing and scalability, immunogenicity risk, and GBM-specific limitations. It is intended to support platform selection according to therapeutic objective rather than to establish a universal hierarchy of delivery systems.

| Characteristic | Albumin-based nanoparticles | Polymeric nanoparticles | Lipid nanoparticles | Viral vectors |
|---|---|---|---|---|
| **Core advantage** | Endogenous protein carrier with hydrophobic drug-binding capacity, albumin-receptor interactions, and clinical precedent for albumin-bound paclitaxel [10,12] | Broad chemical tunability, precision engineering, and controlled-release capability [27] | Efficient encapsulation and delivery of nucleic-acid payloads, especially mRNA and siRNA [71] | High gene-transfer efficiency and strong suitability for gene-replacement or gene-modifying strategies [72] |
| **Biocompatibility** | Generally favorable because albumin is an endogenous plasma protein and has been used in approved biomedical products [10] | Variable depending on polymer chemistry, degradation products, molecular weight, and surface modification [27] | Generally acceptable but formulation-dependent; ionizable lipids, helper lipids, and PEG-lipids can influence tolerability and immune activation [71] | Platform-dependent; immune responses and dose-related toxicities remain important translational concerns [72] |
| **BBB/BTB delivery potential** | May exploit gp60/albondin-mediated endothelial transcytosis, SPARC-associated tumor retention, FcRn-mediated recycling, and caveolin-dependent transport [5,12] | Often requires surface engineering, ligand conjugation, local administration, or BBB-modulation strategies; passive EPR-like delivery is insufficient for many CNS tumors [5,27] | Passive CNS entry is limited; effective brain delivery generally requires active targeting, local administration, or BBB-opening strategies [5,71] | CNS tropism can be engineered, but brain delivery depends strongly on vector type, administration route, tropism, immune response, and safety profile [5,72] |
| **Payload suitability** | Well-suited for hydrophobic small molecules, albumin-binding drugs, selected biologics, and imaging agents [10] | Broad payload compatibility, including small molecules, proteins, nucleic acids, and combination payloads [27] | Particularly suitable for RNA payloads, including mRNA, siRNA, and gene-editing components [71] | Best suited for DNA/RNA expression cassettes, gene replacement, gene editing, and cellular reprogramming strategies [72] |
| **Clinical precedent** | Strong precedent through albumin-bound paclitaxel and other approved albumin-based | Several polymeric nanomedicines have entered clinical development, but translation | Strong clinical precedent for mRNA vaccines and siRNA therapeutics, although GBM- | Increasing clinical precedent in gene therapy, especially AAV-based products, but application |

| | | | | |
|---|---|---|---|---|
| | products; recurrent GBM studies have evaluated albumin-bound paclitaxel with ultrasound-mediated BBB opening [10,14] | remains formulation-specific and affected by reproducibility and scale-up constraints [13,27] | specific CNS delivery remains challenging [71] | to GBM remains modality-, route-, and safety-dependent [72] |
| **Manufacturing and scalability** | Relatively mature for albumin-bound drug products, but product-specific critical quality attributes, albumin conformation, aggregation, sterility, and drug-loading consistency remain essential [10,13] | Scale-up can be difficult because particle size, polydispersity, encapsulation efficiency, and release kinetics are process-sensitive [13,27] | Scalable manufacturing is established for some LNP classes, but formulation reproducibility, storage stability, and cold-chain requirements may remain important constraints [13,71] | Manufacturing is technically complex and requires stringent control of vector yield, potency, purity, empty/full capsid ratio, immunogenicity, and release specifications [72] |
| **Immunogenicity risk** | Generally lower than viral platforms, but aggregation, chemical modification, impurities, or non-human albumin sources may affect immunogenicity [10] | Variable: polymer chemistry, surface charge, degradation products, and targeting ligands may influence immune recognition [27] | Usually manageable, but innate immune activation, complement activation, and anti-PEG responses can occur depending on formulation design [71] | Higher concern relative to non-viral platforms because capsid or viral components can trigger pre-existing or treatment-induced immune responses [72] |
| **Key limitations in GBM** | Delivery remains dependent on BBB/BTB status, albumin-receptor expression, vascular access, and spatial tumor heterogeneity [5,12] | Translation may be limited by heterogeneous tumor penetration, complex optimization, and reproducibility challenges [13,27] | Efficient systemic RNA delivery does not necessarily translate into adequate brain-tumor accumulation without additional BBB-targeting or BBB-opening strategies [5,71] | Best suited for genetic payloads rather than broad small-molecule chemotherapy delivery; safety, tropism, redosing, and manufacturing complexity remain major considerations [72] |
